# Challenges in implementing preventive treatment for latent tuberculosis infection: a narrative review from provider and demand-side perspectives

**DOI:** 10.3389/fpubh.2025.1628218

**Published:** 2025-12-17

**Authors:** Yuxiao Ling, Songhua Chen, Yu Zhang, Qian Wu, Ke Yang, Dan Luo, Yang Li, Yiqing Zhou, Wei Wang, Bin Chen, Jianmin Jiang

**Affiliations:** 1 School of Public Health, Health Science Center, Ningbo University, Ningbo, Zhejiang, China; 2 Department of Tuberculosis Control and Prevention, Zhejiang Provincial Center for Disease Control and Prevention, Hangzhou, Zhejiang, China; 3 Zhejiang Key Lab of Vaccine, Infectious Disease Prevention and Control, Hangzhou, Zhejiang, China; 4 School of Public Health, Hangzhou Medical College, Hangzhou, Zhejiang, China; 5 School of Public Health, Hangzhou Normal University, Hangzhou, Zhejiang, China

**Keywords:** adherence, challenges, latent tuberculosis infection, preventive treatment, review

## Abstract

**Background:**

Preventive treatment is an important measure to interrupt the development of tuberculosis (TB) in people with latent tuberculosis infection (LTBI), which is among the key elements of TB prevention and control in the future. However, the implementation of preventive treatments has been affected by various factors and has fallen far short of expectations. Therefore, this study aims to systematically identify barriers to initiating and implementing LTBI preventive treatment globally from both demand-side and supply-side perspectives, providing targeted evidence to advance the End TB Strategy.

**Methods:**

We systematically searched PubMed and Embase for articles related to preventive treatments. All included articles were peer-reviewed English-language articles published between January 1, 2010, and August 31, 2024. The barriers affecting the initiation and implementation of preventive treatment were extracted from eligible articles and summarized from the two perspectives of supply side and demand side.

**Results:**

Low levels of awareness, concerns about adverse effects, longer treatment periods, and uncertainty regarding the effectiveness of treatment may influence the acceptance of preventive treatment. Furthermore, inadequate knowledge and experience of healthcare workers, limited human and material resources, and low policy priorities may partially hinder the implementation of preventive treatments.

**Conclusion:**

Multidimensional interventions need to be developed to improve acceptance and adherence to preventive treatment and accelerate the implementation. This not only provides direction for future research but also provides a reference basis for clinical practice and policy development to accelerate the process of eliminating TB.

## Background

Latent tuberculosis infection (LTBI) is a dynamic immune system balance that maintains a persistent immune response to the antigenic stimulus of *Mycobacterium tuberculosis* (MTB) after infection. Change to LTBI is not associated with signs, symptoms, imaging findings, or bacteriological evidence of active tuberculosis (TB) (1). The present evidence suggests that LTBI is not contagious. MTB was not eliminated and was in a state of suppression. If the immune system is weakened, dormant MTB may break through immunosuppression, proliferate, and spread throughout the body, which may progress to active TB (2). One-quarter of the global population is estimated to have LTBI, representing a significant reservoir of infection at risk of progression to active TB (1). The numerous patients with LTBI are a potential “patient pool” that continues to deliver new patients with active TB. Approximately 5–15% of people with LTBI would progress to active TB in their future lifetime, with the majority occurring within 2 years of infection (3). The risk of developing active TB may be higher if accompanied by other conditions, such as human immunodeficiency virus (HIV) infection or diabetes (4, 5).

The main goal of preventive treatment is to eliminate MTB present in a resting state in people with LTBI and reduce the possibility of reactivation at a later stage. All individuals who test positive for TB should be examined for signs, symptoms, and bacteriological or imaging results to assess the presence of active pulmonary or extrapulmonary TB before initiating preventive treatment. Preventive treatment is provided after TB has been definitively ruled out. Assuming no ongoing transmission from 2015 onwards, 961 million individuals will be infected by 2035. With a remote LTBI activation rate of 0.15% per year, the incidence of TB in these latent pools in 2035 would be 16.5/100,000 per year, which is higher than the target of the End TB Strategy for 2035 (6). In the absence of other interventions, the incidence of TB could approach 10/100,000 by 2035 if people with LTBI are administered preventive treatment or vaccinated with a new post-infection vaccine. If the treatment of active TB could be combined with the implementation of these interventions, the incidence of TB could be estimated to decrease from 110/100,000 in 2010 (close to the global average in 2010) to 1/100,000 by 2035 (7).

The World Health Organization recommends preventive treatment for people living with HIV, household contacts of patients with bacteriologically confirmed TB, and clinical risk groups. Treatment options include weekly isoniazid and rifapentine for 3 months, daily isoniazid and rifampicin for 3 months, daily isoniazid and rifapentine for 1 month, daily rifampicin for 4 months, and daily isoniazid for ≥6 months (8). Several countries have already reduced the incidence of TB by implementing preventive treatment for people with LTBI (9, 10). In 2018, the United Nations high-level meeting proposed that the number of people on TB preventive treatment should reach at least 30 million globally in 2018–2022; however, only 15.5 million people were provided with preventive treatment, equivalent to 52% of the target (11). A meta-analysis by Rustage et al. showed that the true proportion of preventive treatment initiation among migrants globally was estimated at 69%, and the true proportion of treatment completion was estimated at 74% (12). Bastos et al. observed that 60.4 and 41.9% of people living with HIV initiated and completed preventive treatment, respectively (13). Additionally, the proportion of initiation and completion of treatment among healthcare workers was only 38.5 and 32.0% (14). Considerable challenges and obstacles to the implementation of preventive treatments for LTBI are observed worldwide.

The importance of preventive treatment for LTBI has been widely recognized. However, its implementation effectiveness worldwide falls far short of the expected targets. Although previous studies have identified some barriers, there has been a lack of comprehensive synthesis of challenges from the perspectives of both the demand side (patients) and the supply side (healthcare workers, service systems, and policy support), as well as across the sequential stages of initiation and implementation. In this article, we systematically reviewed publications on the implementation of preventive treatments and explored the difficulties and challenges that may affect the initiation and implementation of preventive treatment from demand-side and supply-side perspectives. This could provide a scientific basis for promoting and enhancing the implementation of TB prevention and control strategies in the later stages.

## Methods

We systematically searched PubMed and Embase databases using Boolean operators (Supplementary table S1). Peer-reviewed English-language articles published between January 1, 2010, and August 31, 2024, including quantitative and qualitative studies related to barriers to or promotion of preventive treatment, were included in the study.

**Figure 1:**
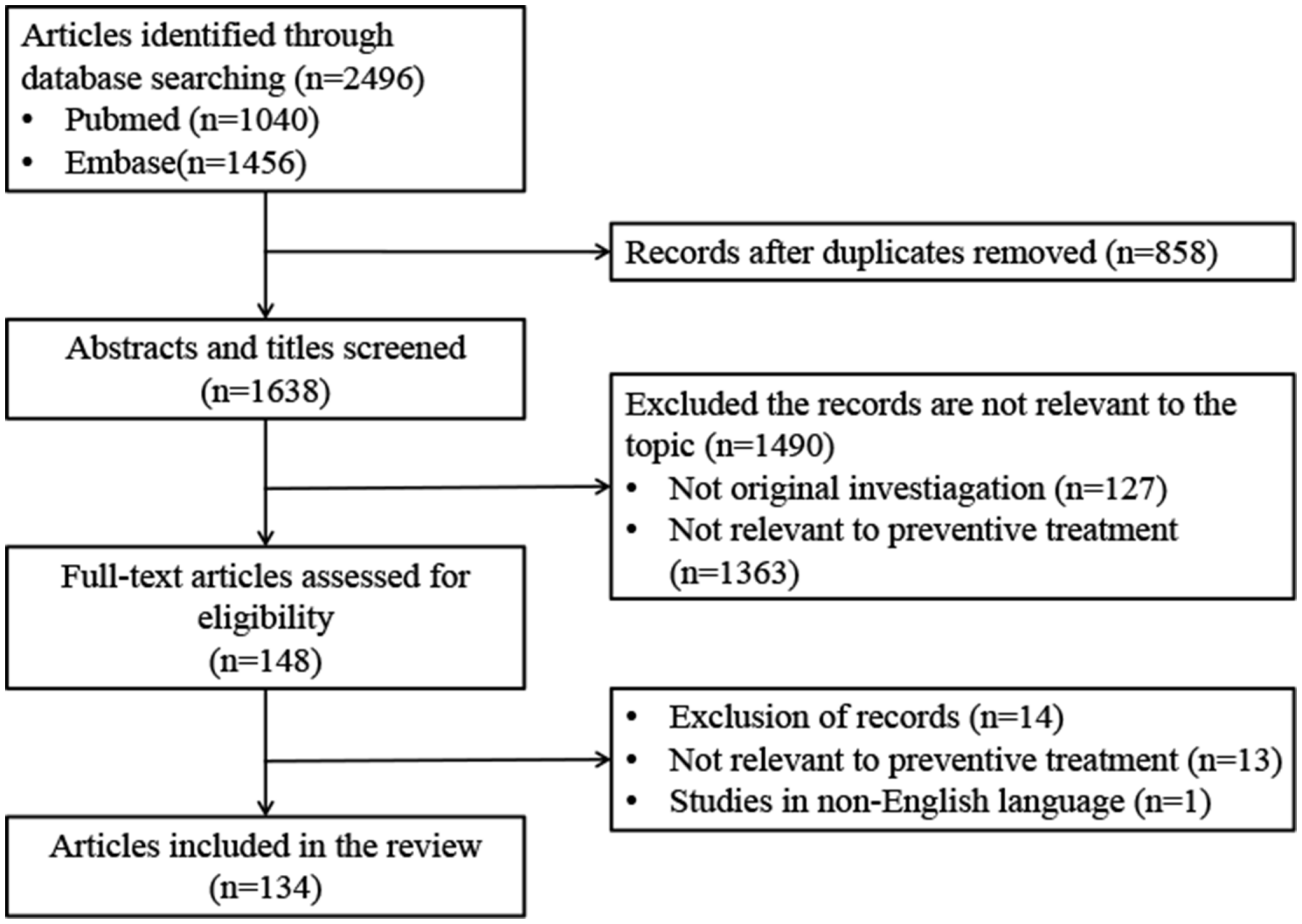
Flowchart for the selection of studies.

This study conducted the literature screening process in accordance with the PRISMA statement (Preferred Reporting Items for Systematic Reviews and Meta-Analyses) (Figure 1). The exclusion criteria for literature screening were as follows: (1) Non-English publications; (2) Non-original research (e.g., conference abstracts, reviews); (3) Literature not directly related to the initiation or implementation barriers of preventive treatment for LTBI. The systematic literature search for this study was fully completed in September 2024, followed by systematic literature screening and data extraction conducted from September to October. And the manuscript was drafted and revised between November 2024 and January 2025. Relevant information on publications that met the criteria was extracted, including the first author, year of publication, study period, study area, study population, study type, proportion of treatment initiation, proportion of treatment completion, and barriers to the initiation and completion of preventive treatment (Supplementary table S2).

We used content analysis to systematically analyze the data from the included articles. Based on the time sequence of TB preventive treatment, the descriptions of potential barriers extracted from the included literature were categorized into two important phases: the initiation phase (encompassing detection and diagnosis) and the implementation phase (encompassing treatment and support). Meanwhile, conduct an in-depth analysis from both the demand-side and supply-side perspectives. For example, patients’ lack of knowledge about preventive treatment and concerns about adverse effects were categorized as barriers in the demand-side initiation phase. The healthcare workers’ lack of knowledge and insufficient resources were categorized as barriers at the supply-side initiation phase. In this way, we comprehensively and systematically identified the potential barriers existing at different stages and levels in preventive treatment. To present the research findings more intuitively, we used a conceptual model to display the analysis results, making the complex research findings clear at a glance and facilitating the readers’ understanding and grasp of key information.

## Results

**Figure 2:**
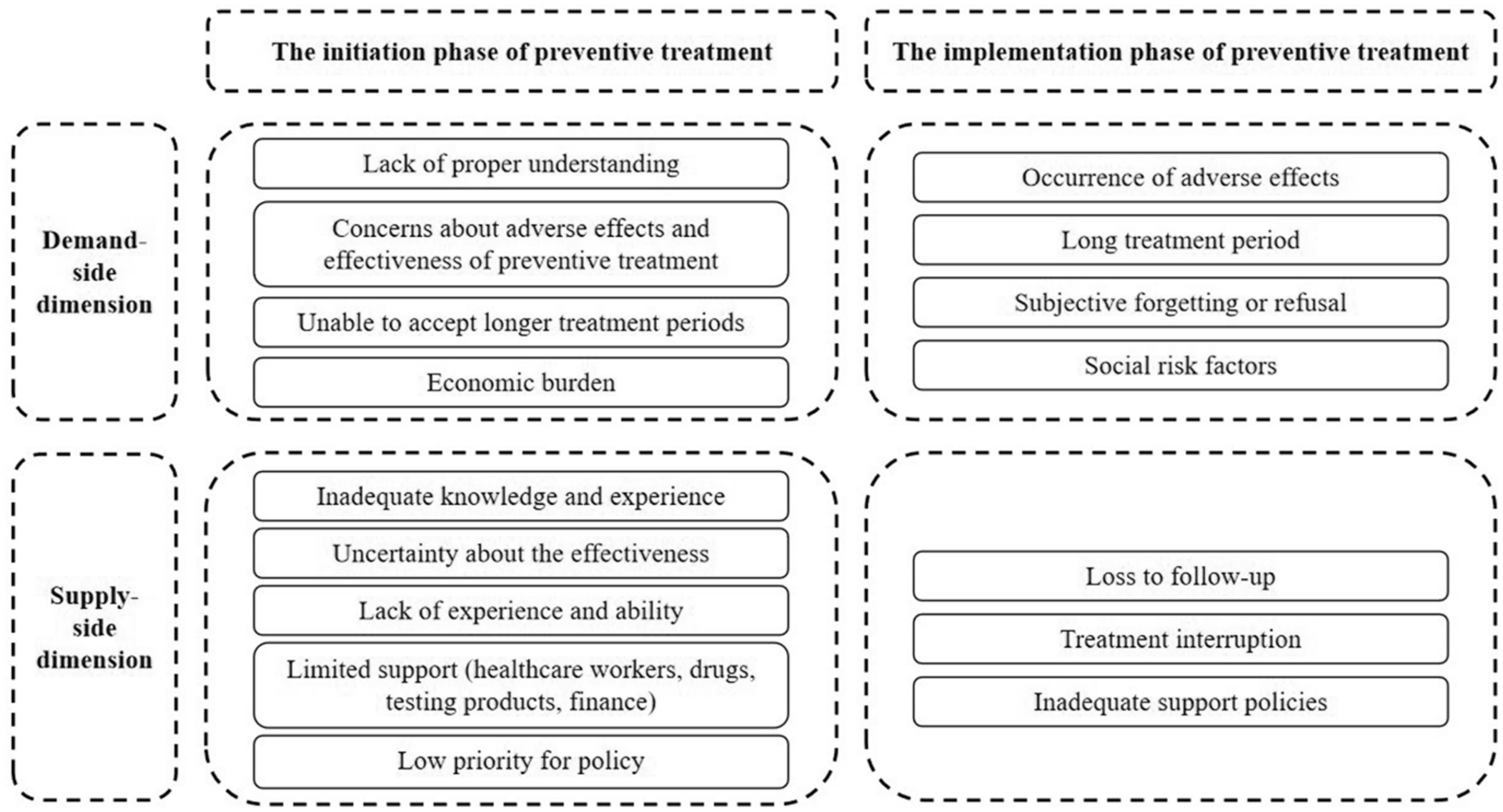
Barriers to the initiation and implementation phases of preventive treatment in literature.

Our study summarized the difficulties and challenges faced during the initiation and implementation of preventive treatment from the demand- and supply-side dimensions and integrated these findings into a conceptual model (Figure 2).

## Challenges in the initiation phase of preventive treatment

### Demand-side dimension

Lower awareness of preventive treatments, fear of adverse effects, length of the treatment period, and uncertainty about the effectiveness of treatment influence the acceptability of preventive treatments in people with LTBI.

#### Lack of proper understanding of LTBI preventive treatment

The most common barrier in the demand-side dimension was inadequate knowledge and understanding of preventive treatment (15). Kunin et al. showed that people with LTBI had low motivation to engage in preventive treatment and had difficulty processing information related to diagnosis (16). A Canadian qualitative study observed a lack of awareness of active TB and LTBI among people with unstable housing or homelessness (17). Illiteracy is associated with not initiating preventive treatment among TB contacts in Brazil (18). Additionally, an inadequate understanding of preventive treatment among parents or caregivers may influence decisions regarding the treatment of children (19). People who do not notice any physical abnormalities may think that they do not have any health problems and refuse to take medication because of a low level of knowledge about LTBI (20–22). Some individuals may feel embarrassed due to the TB-related stigma, believing that family members and friends who know they are being treated will avoid them. This perception may reduce the likelihood of accepting treatment (23).

#### Concerns about adverse effects and effectiveness of preventive treatment

Safety is a major concern in preventive treatment. Individuals’ fears or concerns about potential adverse effects may limit their willingness to initiate preventive treatment (24, 25). Adverse effects included discomfort, fatigue, rash, fever, vomiting, impaired liver function, and impaired renal function. Most first-line anti-TB drugs have a higher probability of adverse events, particularly hepatotoxicity, than other conventional drugs (26). The fear of possible adverse effects could make participants hesitant to undergo preventive treatments. Patients with liver disease or those taking hepatotoxic drugs would refuse preventive treatment after counseling due to adverse drug effects (27). Older adults were less likely to accept treatment than younger adults owing to concerns about hepatotoxicity or other adverse effects (28). The pill burden can also be somewhat stressful to the individual, which can affect the initiation of treatment (17, 29). Some household contacts believed that drugs taken for preventive treatment would make them drug-resistant (22). Parents may refuse treatment for their children out of fear that their development will be affected by the adverse side effects of the medication (19). Additionally, the lack of assurance about the efficacy of preventive treatment is a major barrier to accepting treatment (30). Individuals may be concerned about reinfection from elsewhere even with preventive treatment.

#### Unable to accept longer treatment periods

The treatment process of Preventive treatment is a long-term process for individuals without any manifestations of the disease (22). Patients consider that the influence of life and work at a later stage may cause irregularities in taking drugs, which may lead to the development of drug resistance in the future (31, 32). The cumbersome process of preventive treatment may result in some individuals waiting until the onset of TB before receiving treatment (33).

#### Economic burden

The treatment cost can be significant for some individuals, particularly if they lack health insurance or have limited financing. A multicenter prospective cohort study in Brazil reported that low income was associated with the failure to initiate preventive treatment (18). However, participants with the highest household income were more likely to refuse preventive treatment after initially being willing to accept treatment, whereas individuals from low-income households accepted treatment for fear that developing TB would increase the financial burden on their families (20). Furthermore, unstable living conditions and financial problems associated with unemployment are issues that individuals must consider before starting treatment (17).

### Supply-side dimension

The supply-side dimension of preventive treatment is described in terms of healthcare workers, service systems, and policy support.

#### Inadequate knowledge and experience with preventive treatment and uncertainty about the effectiveness of the treatment

People with LTBI mainly depend on healthcare workers to determine their treatment path because they lack knowledge about LTBI (25). Communicating the necessary health education to people with LTBI is the first step in supporting patients in starting treatment (34). A qualitative study by Szkwarko et al. discovered that most primary care providers and nurses were not knowledgeable about or comfortable with the provision of LTBI treatment. Some participants were unfamiliar with drug-related adverse effects and drug interactions and were unclear about the treatment process and guidelines (15). Healthcare workers have little knowledge about LTBI, its treatment benefits, and the risk of re-exposure to TB in the workplace (32). Additionally, the efficacy of preventive treatments is not guaranteed (30). When the individual benefits outweigh the risks, healthcare workers may consider providing clear treatment recommendations to guide the patient toward preventive treatment (35). When individuals receive negative advice from healthcare providers, they may hesitate to receive treatment. A low level of confidence among providers may result in hesitation to implement preventive treatment programs because of a lack of knowledge and experience with LTBI, concerns about adverse effects, and treatment efficacy.

#### Lack of experience and ability to deal with the occurrence of adverse effects

Many healthcare providers have reported that preventive treatments may cause adverse effects (19). A study in the UK showed that doctors with relevant experience in treating active TB or LTBI were more willing to provide preventive treatment in primary care than inexperienced general practitioners (36). Inexperienced healthcare workers may be unable to develop appropriate preventive treatment plans or may be reluctant to prescribe preventive treatments because of concerns regarding adverse events, medication interactions, or the development of drug resistance (17, 29, 30, 37). Additionally, providers found it challenging to make decisions regarding the appropriateness of treatment for specific populations such as people living with HIV, pregnant women, or patients with other medical conditions (15).

#### Lack of specialized medical staff

Limited human resources are required to perform multiple tasks, including drug distribution, contact tracing, and preventive treatment (22). This may partially increase the workload of healthcare workers, resulting in existing healthcare workers being unable to concentrate on preventive treatment, leading to a lower frequency of prescribing preventive treatment plans (35). A lack of support and training prevents some primary care providers from taking on the additional risks and time associated with LTBI treatment (15, 36).

#### Difficulties in the supply of drugs and testing products

A series of tests necessary to rule out active TB was required before the start of treatment, such as sputum collection, chest radiography, and testing for LTBI. This requires a healthcare system with well-developed laboratory-testing capabilities. The more steps required before treatment, the longer it takes for treatment to begin, and the greater the risk of failure to begin or complete treatment (17). The shortage of funds, inability to accurately determine target quantities, insecure logistical demands, and untimely procurement of medicines may lead to shortages of medicines or test products, affecting the process of implementation of preventive treatment (22, 38). Furthermore, lower accessibility to the health system can limit individuals’ ability to visit healthcare facilities, including the distance from healthcare facilities and difficulty in making treatment appointments (38, 39).

#### Limited financial support

Compared to LTBI, active TB is better funded and delivers more important public health outcomes, such as reduced morbidity and mortality and reduced risk of transmission. Due to policy and economic constraints, funding for healthcare workers, drugs, and equipment is often insufficient to achieve full coverage of all populations requiring screening and treatment (34). Incomplete incentives and protective policies for doctors and patients do not adequately motivate personnel to provide or accept treatment.

#### Low priority for policy

Inertia in health systems and clinical services, and a lack of cost-effectiveness evidence have led to the deprioritization of LTBI (37). LTBI has rarely been prioritized in public health policies and clinical practice, which may affect follow-up management, service delivery, health literacy, and healthcare accessibility. In a survey of 30 high-burden countries by Lena Faust et al., a frequently cited barrier to implementation by respondents was the prioritization of active TB over LTBI management (40). Compared to active TB and other diseases, a lower severity of LTBI leads to a lower prioritization of LTBI treatment. Management of other infectious or chronic diseases in the general population may receive more attention than that of LTBI (32, 37).

## Challenges in the implementation phase of preventive treatment

### Demand-side dimension

#### Occurrence of adverse effects

Several research studies have discovered that the most common reason for individuals to discontinue treatment is the development of adverse drug reactions (17, 41–43). Some participants experienced different levels of adverse reactions to the drugs and stopped taking them according to their own choices (16). Solid organ transplant candidates reportedly discontinued preventive treatment because of hallucinations, peripheral neuropathy, urticaria, nausea or vomiting, and abnormal liver chemistry (44). A retrospective cohort study in Taiwan showed that the most common reason for treatment interruption in long-term care facilities was pharmacological liver injury, followed by the patient’s refusal to continue LTBI treatment and the development of flu-like symptoms (45).

#### Long treatment period

The long treatment period was also one reason why individuals were unable to adhere to treatment completion. Individuals were more likely to complete a short course of treatment than those who received other treatment regimens. A 3-month course of isoniazid combined with rifampicin was more likely to be completed than a 6- or 9-month course of only isoniazid treatment (46–48). Using 6 years of programmatic data, Cambodia observed that individuals on a 3-month daily rifampicin and isoniazid and a 6-month daily isoniazid regimen were more likely to fail to complete treatment than those on a 3-month weekly isoniazid and rifapentine regimen (49).

#### Subjective forgetting or refusal

In a fast-paced social life, individuals may forget to take drugs because of the impact of life and work. Adherence to regular medication can also be a challenge for individuals (50). Individuals may develop inertia when forgetfulness occurs, leading to treatment interruption. Untimely clinic appointments, transportation problems, travel plans, and work conflicts may also contribute to incomplete treatment (43, 51). Furthermore, individuals have the option to interrupt treatment for any reason because of the non-mandatory nature of preventive treatment (48).

#### Social risk factors

Related studies have revealed an association between the age of the individual and completion of treatment; however, this association was unclear. The risk of not completing treatment increased by 0.07 times for each year of increase in age among people living with HIV (52). Age ≥80 years was associated with treatment interruptions among older adults (53). However, studies have also shown that older adults are more likely to complete treatment than those aged < 35 years (14, 28). A lower treatment completion rate was observed in patients younger than 36 years (54). Individuals with poor behavioral habits (such as smoking and drinking) and chronic diseases are also prone to treatment interruption (47, 55, 56). Sex differences may also contribute to differences in treatment completion rates (28, 52, 57, 58). Treatment completion rates are relatively low among immigrant populations (47).

### Supply-side dimension

#### Loss to follow-up and treatment interruption

Loss to follow-up and treatment interruption are the main problems faced by providers during treatment. On the one hand, heavy workloads and staffing constraints make it impossible for healthcare workers to provide additional supervision and reminder services to ensure that individuals take their drugs or visit healthcare facilities on time, which may affect the outcome of the completion of treatment. Changes in the place of residence or work for some special populations could cause healthcare workers to spend more time contacting individuals to assist (17, 43). However, some healthcare workers may resort to interrupting drug administration to mitigate adverse effects because of their limited knowledge and experience.

#### Inadequate support policies

Most countries provide various adherence interventions for patients with TB to enhance management, such as health education and psychoemotional and social support. However, limited support was available for patients receiving LTBI treatment (59). Inadequate government incentives may not adequately motivate healthcare workers and patients to make preventive treatment efforts. Aaldring et al. reported that most countries provided less support to people with LTBI than to patients with TB. Even in countries that provide support to people with LTBI, fewer than half specify adherence interventions for preventive treatment in their guidelines (59).

## Discussion

This review systematically summarized the multifaceted challenges associated with preventive treatment for LTBI documented between 2010 and 2024. The conceptual model constructed views challenges as the outcome of multi-level interacting factors. It advanced existing understanding by clarifying the interplay between patient perceptions, healthcare provider capabilities, and the infrastructure of the healthcare system throughout the treatment process. This provides a more nuanced theoretical basis for developing targeted, multidimensional interventions.

Acceptance of preventive treatment may be limited by insufficient knowledge of LTBI. The treatment attitude of the provider may also somewhat guide individuals. Improving correct awareness and acceptance of preventive treatment in the population are critical steps in implementing preventive treatment. Acceptance of preventive treatment increases when individuals believe that they can transmit MTB or have a better understanding of TB (20, 60, 61). Education on TB and LTBI can be integrated into the overall planning for teaching and health prevention. Public attention to TB and LTBI can be attracted by conducting various forms of popularization activities using multiple social media platforms. Simultaneously, social stigma may be reduced by combining regular LTBI testing with other programs (62).

Increasing the level of awareness of LTBI and understanding the benefits of preventive treatment among healthcare workers were the major contributing factors that enabled them to recommend treatment (63). When confronted with disease counseling, healthcare workers can encourage individuals to accept preventive treatment by adequately explaining it. Furthermore, the clinical professional skills training of healthcare workers should be strengthened to ensure that they have good handling abilities when dealing with adverse effects. Besides, short-course treatment regimens had a higher rate of treatment completion (58, 64). Therefore, short-course treatment regimens should be prioritized for the development of drug regimens. The development of new drugs or vaccines can reduce the risk of active TB and reduce the risk of adverse drug effects.

Enhanced adherence management is an important tool in completing preventive treatment. Implementation of LTBI screening in any setting or population should be followed by intensive adherence management, including further exclusion of TB diagnoses, implementation of preventive treatment, monitoring of adverse effects, and the occurrence of active TB. Considering that individuals have different levels of acceptance of preventive treatments, different management approaches should be adopted. For example, those who accept preventive treatment should focus on medication management to increase the rate of treatment completion. Those who do not accept treatment should strengthen health education and closely observe changes in infection or morbidity status. Patients who maintained their scheduled appointments were 10 times more likely to complete preventive treatment than those who missed one or more appointments (65). Therefore, providing appointment reminder services can somewhat help individuals complete their treatment (43). Adopting dynamic management methods such as regular home follow-up visits and video directly observed therapy also helps to strengthen adherence management and improve the treatment completion rate (58, 66–68).

Increasing the attention of the government and improving the service guarantee system are required. Strengthening financial support is an important solution for LTBI management (34). Individuals can be offered medical benefit policies to encourage them to complete the evaluation and treatment, such as increasing the proportion of medical insurance reimbursements for the cost of chest radiography, incorporating LTBI testing programs into routine screening programs, and providing free drugs. The provision of health insurance was a potential means of changing individual treatment behavior and increasing treatment completion rates (43, 69). Guaranteeing access to healthcare services by increasing the number of primary healthcare facilities and providing convenient outpatient appointments (61). Additionally, developing and implementing incentive policies to motivate healthcare workers to perform treatments is needed. Human resource security should be strengthened through the training and introduction of specialized technical personnel and the recruitment of community members who are well-connected and trusted by the community to participate in management in a coordinated manner.

This study extensively collected journal evidence on the topic through a systematic search and discussed the challenges that may be faced at different stages of preventive treatment from demand- and supply-side perspectives. However, this review had some limitations. First, the results of the studies were not meta-analyzed because of the limitations of the study types. Second, our findings should be interpreted in the context of regional variations in the incidence of TB. In high-burden areas, preventive treatment may face greater prioritization pressures as healthcare resources are primarily directed toward controlling active TB. Low-burden areas may have greater capacity to integrate LTBI screening and treatment into routine services. This disparity underscores the necessity of context-appropriate strategies when implementing LTBI preventive treatment programs. Future research could focus on this regional heterogeneity for more in-depth and targeted studies. Third, our restrictions on the English literature and publication times may have missed some relevant studies. Additionally, the quality of the included studies may have differed. Low-quality studies may have affected our conclusions. Therefore, there is a need to expand the collection scope of study types to obtain a sufficient amount and diverse data. Focus on high-level studies to further explore the barriers and influencing factors in the implementation of preventive treatment. This can provide a more detailed and reliable reference for the development and improvement of preventive treatment management strategies and help to improve the overall level of TB prevention and control.

## Conclusion

Our findings suggest that multiple factors hinder the implementation of preventive treatment. Developing multidimensional interventions to address the challenges faced in preventive treatment, including improving individual understanding and acceptance of preventive treatment, increasing the level of knowledge and ability of healthcare workers, strengthening the management of treatment follow-up, and improving the service system and policy safeguards for preventive treatment is required. This not only provides practical optimization strategies for clinical practice but also provides detailed references for policy formulation and improvement, which will help to further accelerate the process of eliminating TB.

## Data Availability

The original contributions presented in the study are included in the article/Supplementary material, further inquiries can be directed to the corresponding authors.
