# Supplementary material for: Challenges in implementing preventive treatment for latent tuberculosis infection: a narrative review from provider and demand-side perspectives
Source: Front Public Health. 2025 Dec 17;13:1628218. doi: 10.3389/fpubh.2025.1628218 (PMC12753996; doi:10.3389/fpubh.2025.1628218)
Supplement: Supplementary file 1 [file Supplementary_file_1.docx]

**Challenges in Implementing Preventive Treatment for Latent Tuberculosis Infection: A Narrative Review from Provider and Demand-Side Perspectives**

| **Table S1. Search strategy** |
| --- |
| **PubMed** |
| ("Latent Tuberculosis"[tiab] OR "Latent Tuberculosis"[Mesh] OR LTBI[tiab] OR "latent TB"[tiab]) AND (barriers[tiab] OR block[tiab] OR obstacle[tiab] OR hinder[tiab] OR constrain[tiab] OR hard[tiab] OR facilitate[tiab] OR benefit[tiab] OR enablers[tiab] OR attitude[tiab] OR factors[tiab]) AND (treating[tiab] OR treatment[tiab] OR therapy[tiab] OR Intervention[tiab] OR implementation[tiab] OR management[tiab]) AND (("2010"[Date - Publication] : "2024/8/31"[Date - Publication]))  Records = 1040 |
| **Embase** |
| (‘Latent Tuberculosis’:ti,ab OR LTBI:ti,ab OR ‘latent TB’:ti,ab) AND (barriers:ti,ab OR block:ti,ab OR obstacle:ti,ab OR hinder:ti,ab OR constrain:ti,ab OR hard:ti,ab OR facilitate:ti,ab OR benefit:ti,ab OR enablers:ti,ab OR attitude:ti,ab OR factors:ti,ab) AND (treating:ti,ab OR treatment:ti,ab OR therapy:ti,ab OR Intervention:ti,ab OR implementation:ti,ab OR management:ti,ab) AND [2010-2024]/py  Records = 1456 |

| **Table S2. Characteristics of included studies.** | | | | | | | | | |
| --- | --- | --- | --- | --- | --- | --- | --- | --- | --- |
| **First Author** | **Year of publication** | **Study period** | **Study area** | **Study population** | **Study type** | **Proportion of treatment initiation (%)** | **Factors of barriers** | **Proportion of treatment completed (%)** | **Factors of barriers** |
| **Quantitative study** | | | | | | | | | |
| L Anibarro (1) | 2010 | 2004.1-2007.3 | Spain | People with LTBI | Cohort |  |  | 484/599 (80.80) | Age <36 years, male sex, immigrant status <5 years of residence and the presence of social risk factors |
| Asuncion Diaz (2) | 2010 | 2000.3-2003.2 | Spain | People living with HIV | Cohort | 122/181 (67.40) |  | 70/122 (57.38) |  |
| Ingunn Harstad (3) | 2010 | 2005.1-2006.6 | Norway | Asylum seekers | Cohort | 30/2237 (1.34) |  |  |  |
| C Robert Horsburgh Jr (4) | 2010 | 2002 | United States and Canada | People with LTBI | Cross-sectional study | 597/720 (82.92) |  | 1045/1994 (52.41) | Starting the 9-month isoniazid regimen, residence in a congregate setting (nursing home, shelter, or jail), injection drug use, age > or= 15 years, and employment at a health-care facility |
| Jiehui Li (5) | 2010 | 2002.1-2004.8 | United States | People with LTBI | Cohort |  | Social situation, medical contraindication, patient’s refusal | 6788/15035 (45.15) |  |
| Neela D Goswami (6) | 2012 | 2008.1-2009.5 | United States | People with LTBI | Cohort | 130/496 (26.21) | The patient’s lack of understanding of the disease, specialists were reluctant to diagnose and treat | 70/130 (53.85) |  |
| Janine Young (7) | 2012 | 2006.11-2009.3 | United States | Immigrants | Cohort |  | Health-care facilities | 121/150 (80.67) | Older age, increased number of days between TST and CXR, and 0–1 well care visits before TST placement |
| P W Colson (8) | 2013 | 2007.3-2008.9 | United States and Canada | People with LTBI | Cross-sectional study | 1515/1692 (89.54) |  |  |  |
| B Kan (9) | 2013 | 2002.3-2007.12 | Sweden | People with LTBI | Cohort |  |  | 272/360 (75.56) | Younger patients, patients originating from Somalia and asylum seekers |
| Saiyud Moolphate (10) | 2013 | 2012.7-2012.9 | Thailand | Healthcare workers | Cross-sectional study |  |  |  |  |
| Jamie P Morano (11) | 2013 | 2003.1-2011.7 | United States | People with LTBI | Cohort | 135/307 (43.97) | Health care worker, being previously recommended for treatment and believing that taking medicines would be problematic | 75/135 (55.56) |  |
| April C Pettit (12) | 2013 | 2007.3-2008.9 | United States and Canada | People with LTBI | Cohort |  |  | 617/1306 (47.24) | Female sex and current alcohol use |
| Shiow-Huey Chang (13) | 2014 | 2005.1-2011.8 | United States | People with LTBI | Cross-sectional study |  | Unclear direction of national policy, fear of emerging Isoniazid resistant tuberculosis, and fear of poor adherence | 1235/1587 (77.82) | Age 15-18 years, non-Hispanic race, development of hepatitis, and symptoms of adverse effects |
| C T Fiske (14) | 2014 | 2002.1-2006.12 | United States and Canada | Close contacts | Cohort |  |  | 723/1147 (63.03) | Study site, initial interview sites other than a home or health care setting and isoniazid preventive treatment |
| Kathy Malejczyk (15) | 2014 | 2005.1-2010.12 | Canada | People with LTBI | Retrospective study |  |  | 57/77 (74.03) | Homelessness |
| Aman Sidhu (16) | 2014 | 2001.1-2010.12 | Canada | Solid organ transplant Candidates | Retrospective study |  |  | 122/189 (64.55) | Patients who were liver transplant candidates |
| Daniela Silva de Aquino (17) | 2015 | 2007.11-2012.5 | Brazil | People living with HIV/AIDS | Cohort | 162/232 (69.83) |  |  |  |
| H Bishara (18) | 2015 | 2005-2011 | Israel | Immigrants | Cohort |  |  | 628/663 (94.72) | Side effects |
| Y Hirsch-Moverman (19) | 2015 | 2007.3-2008.9 | United States and Canada | People with LTBI | Cross-sectional study |  |  | 617/1323 (46.64) | Severe symptoms, the inconvenience of clinic/pharmacy schedules, barriers to care and changes of residence, patient concerns about tolerability/toxicity, appointment conflicts, low prioritization of TB, and forgetfulness |
| Jennifer B Nuzzo (20) | 2015 | 2009.2-2011.3 | United States | Refugees and other patient groups | Retrospective study | 652/841 (77.53) | Only treatment in one of the two referral services | 495/652 (75.92) |  |
| Angela Marcia Cabral Mendonça (21) | 2016 | 2002.1-2009.12 | Brazil | Children and Adolescents | Cohort |  |  | 183/245 (74.69) | Human Development Index of the place of residence and the contact with adults |
| E I Okoli (22) | 2016 | 2010.7-2011.11 | South Africa | People living with HIV | Cohort |  |  | 66/104 (63.46) | Drug out of stock, loss to follow-up and Drug not prescribed |
| Vidya Pathak (23) | 2016 | / | Australia | Healthcare workers | Cross-sectional study | 64/106 (60.38) | Female |  |  |
| Elissa Rennert-May (24) | 2016 | 2009.1-2011.12 | Canada | Refugees | Cohort |  |  | 103/141 (73.05) | Young age and Sub-Saharan African origin |
| Jaime K Walters (25) | 2016 | 2009.11-2012.10 | United States | Refugees and other patient groups | Cohort |  |  | 261/381 (68.50) | Refugees from Middle East and North African birth countries |
| Asnakew Achaw Ayele (26) | 2017 | 2016.3-2016.6 | Ethiopia | People living with HIV/AIDS | Cross-sectional study |  | Monitor yourself for signs and symptoms, side effects, treatments that are unnecessary, have been vaccinated | 139/154 (90.26) | Carelessness and forgetfulness, side effects, being away from their residential areas or homes |
| McKenna C Eastment (27) | 2017 | / | United States | People with LTBI | Cohort |  |  | 259/393 (65.90) | Unmarried, used tobacco and/or alcohol, and had more medical problems, received charity care |
| Lisa Kawatsu (28) | 2017 | 2007-2014 | Japan | People with LTBI | Longitudinal study |  |  | 20184/33156 (60.88) | Aged 65 years and above, foreign-born, healthcare professional, full-time and part-time worker and detected via contact investigation |
| Amy L Sandul (29) | 2017 | 2011.6-2013.12 | United States | People with LTBI | Cohort |  |  | 2793/3288 (84.95) | Incarceration and homelessness |
| Francesca Bonvicini (30) | 2018 | 2012.1-2013.12 | Itali | Immigrants | Cross-sectional study |  |  | 14/28 | Refugees, higher education levels |
| Huaping Huang (31) | 2018 | 2014.12 | China | Staff and students | Cross-sectional study |  |  | 69/362 (19.06) | Discrimination, worry about adverse drug reactions, a low parental education level, and accepting the opinion of a non-TB specialist |
| Miranda G Loutet (32) | 2018 | 2014.8-2015.8 | England | Immigrants | Cohort | 449/616 (72.89) |  |  |  |
| M M Macaraig (33) | 2018 | 2015.1-6 | United States | People with LTBI | Retrospective study |  |  | 313/449 (69.71) | Refusal to continue treatment |
| O Olsson (34) | 2018 | 2008.1-2016.3 | Sweden | Immigrants | Retrospective study |  |  | 506/606 (83.50) | Immunosuppressive treatment, treatment started after 30th June 2013 |
| Yvette Louise Schein (35) | 2018 | 2016.1-2016.12 | Norway | People with LTBI | Cohort |  |  | 658/726 (90.63) | Adverse events |
| Michael Scolarici (36) | 2018 | 2010.1-12 | United States | People with LTBI | Cohort |  |  | 69/114 (60.53) | Loss to follow-up, elevated liver enzymes, cost/access, minor side effects, rash, drug interactions and clinical decision to stop. |
| Erica L Stockbridge (37) | 2018 | 2011.7-2014.3 | United States | People with LTBI | Cross-sectional study |  |  | 495/1072 (46.18) | Age, patient location |
| Susannah K Graves (38) | 2019 | 2013 | Mozambique | Healthcare workers | Cross-sectional study | 234/333 (70.27) |  | 181/234 (77.35) | Gastrointestinal side effects, abandoned treatment |
| Amos Lal (39) | 2019 | 2006.12-2010.10 | United States | Persons Seeking Immigration and Health Care Workers | Retrospective study |  |  | 88/128 (68.75) | Health care workers |
| Hyun Lee (40) | 2019 | 2016.11-2017.3 | Korea | Healthcare workers | Cohort | 128/189 (67.72) |  |  |  |
| Christopher Pease (41) | 2019 | 2012.1-2016.3 | Canada | People with LTBI | Cohort | 246/328 (75.00) |  | 186/246 (75.61) | Older age |
| Pierre J Plourde (42) | 2019 | 1999.4-2014.3 | Canada | People with LTBI | Cohort |  |  | 3319/5514 (60.19) | Older age, INH270, and RIF120 |
| Marie Nancy Séraphin (43) | 2019 | 2009-2015 | United States | People with LTBI | Cohort |  | Having a liver disease or currently taking hepatotoxic drugs, being a physician, other patient-related healthcare workers and years of employment ≥20 years | 6201/12495 (49.63) | Black patients, Hispanic patients, and non-U.S.-born persons living in the United States ≤5 years |
| Simone Villa (44) | 2019 | 1992.1-2018.12 | Italy | People with LTBI | Retrospective study |  | Older age and undergoing TST due to employment screening | 14197/17859 (79.49) | Homeless |
| Nélia C N Araújo (45) | 2020 | 2009-2014 | Brazil | Contacts | Cohort |  |  | 249/300 (83.00) | Age |
| Sung Jun Chung (46) | 2020 | 2016.6-2017.12 | Korea | Healthcare workers | Cohort |  |  | 71/114 (62.28) | Hepatotoxicity |
| Jia-Yih Feng (47) | 2020 | 2017.1-2019.5 | China | People with LTBI | Cohort |  |  | 332/406 (81.77) | The 9H regimen, age ≥80 years, and systemic adverse reactions |
| Haftom Legese (48) | 2020 | 2019.4-2019.8 | Ethiopia | People living with HIV | Cross-sectional study |  |  | 321/372 (86.29) |  |
| Adoma Manful (49) | 2020 | 2012.1-2016.12 | United States | Refugees | Cohort | 690/1208 (57.12) |  | 432/690 (62.61) | Individuals who were screened earlier |
| Fayette Nguyen Truax (50) | 2020 | 2010.1-2011.8 | United States | People with LTBI | Cohort | 303/474 (63.92) |  | 220/282 (78.01) | Medication side effects, transportation issues and conflicts with travel plans or work schedules |
| Simon Sensalire (51) | 2020 | 2019.1-2019.6 | Uganda | People living with HIV | Cohort |  |  | 901/1010 (89.21) |  |
| Alexis Sentís (52) | 2020 | 2013-2017 | Portugal | People with LTBI | Cohort |  | Older age, living further away from the TB clinic and having entered the country in a family of 2 or 3 | 13346/15478 (86.23) | Older than 15 years, being born abroad for (Asia, Africa), having a chronic disease, alcohol abuse, and being intravenous drug user, three-month course treatment with isoniazid plus rifampicin |
| Yoshiyuki Sugishita (53) | 2020 | 2014.1-2014.12 | Japan | People with LTBI | Cross-sectional study |  |  | 877/1060 (82.74) | Side effects, could not be followed up |
| Melanie D Swift (54) | 2020 | 2006.1-2015.12 | United States | Healthcare workers | Cohort | 193/470 (41.06) |  | 137/193 (70.98) |  |
| Maskit Bar-Meir (55) | 2021 | 2000-2015 | Israel | Healthcare workers | Cohort | 60/124 (48.39) | Drug side effects, emergence of drug resistance and the likelihood of reinfection |  |  |
| Sivaporn Gatechompol (56) | 2021 | 2018.8-2019.11 | Thailand | Prisoners | Cross-sectional study |  |  | 356/383 (92.95) | Side effects of INH |
| Shareen A Iqbal (57) | 2021 | 2005.1-2016.12 | United States | People with LTBI | Cohort |  | Age of the healthcare worker, occupation of clinician or researcher | 10725/21510 (49.86) | Those younger than 45 years, living in the Northeast or South Census regions, and women |
| K Sullivan (58) | 2021 | 2010.1-2016.12 | Canada | People with LTBI | Retrospective study | 1203/1771 (67.93) | Did not recall receiving a clear medical recommendation, PPD interpreted as borderline or false positive, offered chest X-ray follow-up instead of LTBI treatment, protection of previous BCG immunization, side effects or length of treatment, low TB risk | 795/1203 (66.08) | Referral from the TB Clinic back to the primary care team following initial consult and treatment with the standard of 9 months of Isoniazid |
| E Van Ginderdeuren (59) | 2021 | 2015.8-2016.8 | South Africa | Contacts | Cohort |  |  | 24/46 (52.17) |  |
| Chien-Hsing Wu (60) | 2021 | 2018.9-2019.9 | China | End-stage renal disease patients | Cohort |  |  | 50/75 (66.67) | Lower proportion of serum albumin level < 4.0 g/dL |
| Hani S Almugti (61) | 2022 | / | Saudi Arabia | Healthcare workers | Cross-sectional study | 44/60 (73.33) | Older age and female gender | 20/44 (45.45) | The long duration of treatment, and being the treatment optional |
| Ting-Fang Chiu (62) | 2022 | 2017.5-2020.9 | China | Residents and employees of long-term care facilities | Cohort |  |  | 212/264 (80.30) | Drug-induced liver injury, patients’ refusal to continue LTBI treatment, and development of flu-like symptom |
| Lifeng Fa (63) | 2022 | 2020.11-202.12 | China | Healthcare Workers | Cross-sectional study | 4657/4796 (97.10) | Worrying about acquired drug resistance, worrying about adverse events, unguaranteed efficacy, lacking financial support, lacking sufficient staff, troublesome administration of medication, no policy support |  |  |
| Rebecca Helena Holmes (64) | 2022 | 2010.1-2016.12 | Australia | Children and adolescents | Cohort |  | Low knowledge about some clinical facts of LTBI | 296/402 (73.63) | Side effects |
| Samuel B Holzman (65) | 2022 | / | United States | People with LTBI | Cohort | 1073/2515 (42.66) |  | 817/1073 (76.14) | Hispanic ethnicity and homelessness |
| Kuan-Yin Lin (66) | 2022 | 2019.7-2021.12 | China | People living with HIV | Cohort | 309/420 (73.57) | Drug resistance, adverse events, and unguaranteed efficacy | 284/309 (91.91) | Older age, female, anti-HCV positivity, and higher plasma HIV RNA |
| Jinsoo Min (67) | 2022 | 2017.3-2018.12 | Korea | Employees of hospitals and healthcare centers | Cohort | 17500/45485 (38.47) |  | 14572/17500 (83.27) | Radiology technologist, Laboratory technologist, people visited public health center, 9-month isoniazid monotherapy |
| Jinsoo Min (68) | 2022 | 2017.3-2018.12 | Korea | First-grade high school students, employees of educational institutions, employees of social welfare facilities | Cohort | 33500/48865 (68.56) | Age > 65 years, homelessness, evaluation for student/employment clearance, and having discordant test results, persons of Asian race, those evaluated as part of contact investigation, and those tested by IGRA | 27875/33500 (83.21) | Male, age, small to medium-sized city, Moderate high income, public health centres, type of initial regimen, charlson comorbidity index ≥ 3 |
| Tessa Runels (69) | 2022 | 2012.9-2014.11 | United States | People living with HIV and those who use substances | Cohort | 5/11 (45.45) | Physicians’ concerns and patients’ refusal | 3/5 (60.00) | Side effects |
| Nandini Sharma (70) | 2022 | 2020.6-2020.11 | India | Household Contacts | Cross-sectional study | 394/536 (73.51) | Male, age, laboratory technologist, people visited public health center |  |  |
| Luciana Sobral (71) | 2022 | 2015.8-2019.7 | Brazil | Close contacts | Longitudinal study | 139/291 (47.77) | Male, aged ≥ 65 years, city, moderate high and high income, public health centres | 65/139 (46.76) | Younger age of contacts and TB index cases who were female, had pulmonary cavities, and persistent cough |
| Luciana Sobral (72) | 2022 | 2015.8-2019.7 | Brazil | Close contacts | Longitudinal study | 492/933 (52.73) |  | 252/454 (55.51) | Race black or pardo and age |
| Victor Abiola Adepoju (73) | 2023 | 2020.7-2020.9 | Nigeria | Children | Cross-sectional study |  | Per-capita income > 30,000 and perceived TB Severity score ≤ 6 | 155/262 (59.16) |  |
| Katia J Bruxvoort (74) | 2023 | 2009-2018 | United States | People with LTBI | Cohort | 22422/26141 (85.77) |  | 14644/22422 (65.31) | Race, individuals born in a high-TB incidence country, rifampin and other regimens |
| J Cochran (75) | 2023 | 2016.10-2019.3 | United States | Immigrants | Cohort |  | Lower income, illiteracy and secondary smoking | 645/1368 (47.15) | Loss to follow-up, medication side effects, or patient choice |
| Susan Levine (76) | 2023 | 2019.2-2019.7 | United States | People with LTBI | Cross-sectional study |  |  | 33/35 (94.29) |  |
| N Ortiz Laza (77) | 2023 | 2003-2022 | France | Contacts | Cohort |  | Individuals aged ≥35 years, individuals born in a high-TB incidence country | 2048/3066 (66.80) |  |
| Trevor M Stantliff (78) | 2023 | 2019.1-2020.12 | United States | People with LTBI | Cohort |  |  |  |  |
| Luan Nguyen Quang Vo (79) | 2023 | 2019.5-2020.9 | Viet Nam | Household contacts, vulnerable community members and healthcare workers | Cohort |  |  | 892/1107 (80.58) | The 3HR regimen and healthcare workers |
| H Yang (80) | 2023 | 2020.9-2020.12 | Korea | Healthcare workers | Cross-sectional study | 60/110 (54.55) |  | 34/60 (56.67) | No confident, side effects, self-determination, a lot of work, inconvenience |
| Yemin Yuan (81) | 2023 | 2020.7-2020.11 | China | College students | Cross-sectional study | 287/723 (39.70) | Age, healthcare worker |  |  |
| Yom An (82) | 2024 | 2018-2023 | Cambodia | People with LTBI | Cross-sectional study |  |  | 13963/14262 (97.90) | 15-24 and 25-34 years old, individuals initiated with 3-month daily Rifampicin and Isoniazid or with 6-month daily Isoniazid, those who began TPT at referral hospitals |
| Dieudonné Bidashimwa (83) | 2024 | 2004-2020 | Democratic Republic of the Congo | People living with HIV | Cohort |  | Smokers and had a longer work experience in healthcare facilities, a lot of work, side effects, inconvenience, no need | 3457/5625 (61.46) | Health zone of residence and type of antiretroviral therapy regimen |
| Hui Chen (84) | 2024 | 2018.1-2020.8 | China | Close contacts | Cross-sectional study |  | Father’s education level, household income | 2121/2434 (87.14) | Adverse effects |
| Chia-Yu Chiu (85) | 2024 | 2010.1-2023.6 | United States | Solid organ transplant Candidates | Retrospective study |  |  | 457/473 (96.62) | Adverse events, which included hallucination, peripheral neuropathy, urticaria, nausea/vomiting, and abnormal liver chemistries |
| Senedu Bekele Gebreegziabher (86) | 2024 | 2020.4-2022.3 | Ethiopia | People living with HIV | Retrospective study |  |  | 507/719 (70.5) | Health facilities |
| Adam Gray (87) | 2024 | 2021.10-2022.8 | United Kingdom (UK) | People with LTBI | Cohort | 22/35 (62.86) |  | 17/22 (77.27) | Hazardous alcohol use and "competing priorities" |
| Yejin Kim (88) | 2024 | 2016.1-2020.12 | Korea | People with LTBI | Retrospective study |  |  | 6848/11362 (60.27) | 9 months of isoniazid regimen prescription, management in a primary hospital, a hospital’s location in a non-metropolitan region, and management by a non-pediatric specialist |
| Pedro Plans-Rubió (89) | 2024 | 2019.1-2021.6 | Spain | Contacts | Cross-sectional study |  |  |  | Exposure at school or workplace, exposure to an index case without laboratory confirmation of TB, immigrant contact, male gender and exposure duration < 6 h per week or sporadic |
| Alebachew Zewdu Tegegnework (90) | 2024 | 2022.6-2022.7 | Ethiopia | People living with HIV | Cross-sectional study |  | Competing priorities (e.g. currently sleeping on the street), participant concerns about impact from rifampicin on methadone dose stability, and long duration of the rifampicin-free regimen, low potential individual benefit | 484/581 (83.30) |  |
| Yemin Yuan (91) | 2024 | 2020.9 | China | College students | Cross-sectional study | 723/1547 (46.74) |  |  |  |
| Wan Muhammad Zainol Zakaria (92) | 2024 | 2023.5 | Malaysia | Healthcare workers | Cross-sectional study | 137/256 (53.52) |  |  |  |
| Jeffrey I Campbell 39238842 (93) | 2024 | 2018.1- 2019.12 | United States | Pregnant individuals | Cohort | 32/61 (52.46) | breastfeeding, still or newly pregnant, and attempting to conceive | 15/32 (46.88) |  |
| Michael Asare-Baah 38915338 (94) | 2024 | 2016.1-2017.10 | United States | People with LTBI | Cohort | 993/1838 |  | 723/993 | adverse event, male, substance misuse, homelessness or incarceration |
| **Qualitative study** | | | | | | | | | |
| Paul W Colson (95) | 2010 | 2001-2004 | United States | People with LTBI | Qualitative studies | Knowledge of TB, attitudes towards TB/LTBI |  |  |  |
| Linda Hill (96) | 2010 | / | United States | High school students | Qualitative studies | Competing priorities, transportation problems and financial constraints |  |  |  |
| Mark L Wieland (97) | 2012 | 2008.10-2009.1 | United States | Immigrants and refugees | Qualitative studies | Suspicion of generic medications and perceived side effects |  |  |  |
| Kalpana Babu (98) | 2013 | / | India | Healthcare workers | Qualitative studies | The higher cost, limited interpretation of results, the inability to differentiate active and latent TB, and technical issues related to the test |  |  |  |
| Niesje Jansen-Aaldring (99) | 2018 | 2016.6 | 19 European low tuberculosis incidence countries | 19 European low tuberculosis incidence countries | Qualitative studies | Patient support |  |  |  |
| Claudia C Dobler (100) | 2018 | / | Australia | TB physicians | Qualitative studies | Explaining the concept, providing guidance, framing treatment decisions as a choice, the risk of developing TB, adverse effect, unwarranted treatment variation |  |  |  |
| L C Berrocal-Almanza (101) | 2019 | / | England | Community, community-based organisations and public sector stakeholders | Qualitative studies | Capacity of service providers, collaboration between stakeholders, migrant cultures and trust between migrants and service providers |  |  |  |
| Deborah A Milinkovic (102) | 2019 | 2017.8-11 | Canada | Health system advisors and planners, providers, and clients of LTBI health services | Qualitative studies | Low prioritization of LTBI |  |  |  |
| Chris Degeling (103) | 2020 | / | Australia | Immigrants | Qualitative studies | Stigmatize participants and that poor awareness of LTBI |  |  |  |
| Leila Barss (104) | 2020 | / | Ghana | People with LTBI and health care workers | Qualitative studies | Lack of adequate knowledge |  |  |  |
| Lena Faust (105) | 2020 | 2019.5 | 30 TB high-burden countries | 30 TB high-burden countries | Qualitative studies | Shortage of products (eg, PPD), high costs (eg, IGRAs), and lack of regulatory approval of newer treatments (eg, rifapentine) |  |  |  |
| Julie Hall (106) | 2020 | / | Australia | Immigrants | Qualitative studies | Lack of understanding of LTBI and sensitivity to being “targeted” |  |  |  |
| Benissa E Salem (107) | 2020 | / | United States | Homeless persons | Qualitative studies | Lack of LTBI treatment readiness, substance use |  |  |  |
| Nandini Sharma (108) | 2020 | 2019.1 | India | Medical officers, resident doctors, medical interns | Qualitative studies | Medication side effects |  |  |  |
| Amber Heyd (109) | 2021 | 2019.6-2020.1 | Canada | Unstably housed or homeless individuals | Qualitative studies | Competing priorities, difficulty in reaching clients, undesirable aspects of the regimen and difficulties obtaining and initiating 3HP |  |  |  |
| M Saiful Islam (110) | 2021 | / | Seven high tuberculosis burden countries | Eleven key-stakeholders | Qualitative studies | Poor infrastructure, inadequate space for isolation, lack of TB IPC training, limited supply of personal protective equipment, the discomfort of using N95 respirators, and a high number of TB patients |  |  |  |
| Ruvandhi R Nathavitharana (111) | 2021 | / | South Africa | Nurse and physician stakeholders | Qualitative studies | LTBI knowledge gaps, safety and cost concerns |  |  |  |
| Mercedes Yanes-Lane (112) | 2021 | 2019.5-2019.7 | Brazil | Healthcare workers, household contacts and index-TB patients | Qualitative studies |  |  |  |  |
| Adam Thorburn Gray (113) | 2022 | 2022.2-2022.4 | United Kingdom (UK) | Healthcare professionals | Qualitative studies | Lack of resource, and the complexity of clinical decision, Low prioritization of LTBI |  |  |  |
| Gail Gustavson (114) | 2022 | 2019.10-2020.6 | United States | People with LTBI | Qualitative studies | Understanding of LTBI |  |  |  |
| Daria Szkwarko (115) | 2022 | / | United States | Primary care providers and nurses | Qualitative studies | Participants felt least confident with LTBI treatment and held misconceptions about LTBI testing, such as high cost |  |  |  |
| Lifeng Fa (63) | 2022 | 2020.11-202.12 | China | Healthcare Workers | Cross-sectional study |  |  |  |  |
| Yom An (116) | 2023 | 2020.10-2020.12 | Cambodia | Healthcare workers | Qualitative studies | TPT side effects, poor adherence to TPT, poor understanding of TPT among caregivers, TPT risk perception among caregivers, TPT’s child-unfriendly formula, TPT supply-chain issues, caregivers’ concern about the effectiveness of TPT, being non-parental caregivers, and poor community engagement |  |  |  |
| Jessica Carter (117) | 2023 | / | United Kingdom (UK) | Healthcare workers | Qualitative studies | Overly complex pathways that required extensive administrative/clinical time and lacked financial/expert support |  |  |  |
| Siti Nur Farhana H (118) | 2023 | 2019.7-2021.1 | Malaysia | Healthcare workers | Qualitative studies | Uncertainties about the need for LTBI treatment, the attitude of the treating physician towards treatment, time constraints during clinical consultations, and concerns about the treatment itself |  |  |  |
| Anusha Manoharan (119) | 2023 | 2020.7-2020.12 | Malaysia | People with LTBI | Qualitative studies | Misinterpretation of TST, inadequate explanation of TST, and apprehensions about potential medication side effects |  |  |  |
| Isadora Salles (120) | 2023 | 2019.12-2020.2 | Brazil | People living with HIV and their health care providers | Qualitative studies | Financial and food insecurity, difficulties leaving work for appointments, stigma and family responsibilities |  |  |  |
| Isdore Chola Shamputa (121) | 2023 | 2020.10-2020.11 | Canada | Immigrants | Qualitative studies | Lack of knowledge about TB, perceived financial barriers, perceived limited healthcare access, and stigma |  |  |  |
| Brigitte C Spence (122) | 2023 | 2020 | United States | People with LTBI | Qualitative studies | Lack of support, uncomfortable side effects, and pervasive minimization of the positive impact of treatment on their health |  |  |  |
| Prathiksha Giridharan (123) | 2024 | 2023.2 | Eight South East Asian Region countries | Eight South East Asian Region countries | Qualitative studies | Poor demand and low level of acceptance by the beneficiary, low level of acceptance to provide TPT among the providers, challenges in ruling out active TB, issues with supply and supply chain management of diagnostic tests and drugs |  |  |  |
| **Mixed-method study** | | | | | | | | | |
| Christina Atchison (124) | 2015 | 2014.2-2014.4 | United Kingdom (UK) | General practitioners | Mixed-method study | / | Insufficient experience among GPs of screening and treating LTBI, lack of timely specialist support and lack of allied healthcare staff |  |  |
| C-L Walker (125) | 2018 | / | England | Immigrants | Mixed-method study | / | Loss of reputation and stigma |  |  |
| Nwabunie Nwana (126) | 2019 | 2011.7-2015.6 | United States | Homeless persons | Mixed-method study |  |  | 301/393 (76.59) | Staffing and time requirements for treatment, pill burden, higher costs, and difficulty in following up |
| Ineke Spruijt (127) | 2019 | 2016.3-2016.9 | Netherlands | Immigrants and TB physicians | Mixed-method study | 49/94 (52.13) | No perceived advantages of LTBI treatment by the client, Return of client to country of origin in foreseeable future, objection against long duration of PT / afraid of side-effects, dubious IGRA value | 34/49 (69.39) | Interruption of treatment (Side-effects, Pregnancy), Difficulties with follow-up appointments, Difficulties with duration of LTBI treatment, Not understanding the difference LTBI and active TB, False-diagnosis of MDR-TB, Problems within family: difficulty reaching the client for follow-up |
| Ineke Spruijt (128) | 2019 | 2016.11-2017.12 | Netherlands | Asylum seekers | Mixed-method study | 149/178 (83.71) | Having to travel to public health services, language barriers and moving from asylum seeker centres to the community | 129/149 (86.58) | Did not know how to reschedule appointments and had financial or practical difficulties |
| Serah Kajuju Ngugi (129) | 2020 | 2008.1-2017.12 | Kenya | People living with HIV | Mixed-method study | 152/225 (67.56) | Had not been informed that needed to be on IPT, declined the treatment, pill burden and the other fear of side effect | 94/115 (81.74) | Fear of adverse drug reaction, pill burden and lack of an integrated monitoring and evaluation system for IPT |
| Ineke Spruijt (130) | 2020 | / | Netherlands | Participants, key figures, and TB staff | Mixed-method study | 29/30 (96.67) |  | 28/29 (96.55) | Competing priorities in the target group, perceived good health, poor risk perception, and scepticism towards the program purpose |
| Marina Kunin (131) | 2022 | 2017.1-2018.4 | Australia | People with LTBI | Mixed-method study |  |  | 23/31 (74.19) | Adverse reaction, opting out and relocation |
| Claudyne Chevrier (132) | 2023 | 2015.1-2020.8 | Canada | Refugees, clinic staff | Mixed-method study |  |  | 138/230 (60.00) | Treatment regimen |
| Yasir Alvi (133) | 2024 | 2022.1-2022.6 | India | Household Contacts | Mixed-method study | 5571/7309 (76.22) | Apprehension about taking TPT, erratic drug supply, long duration of treatment, side effects, overburden, large population, INH resistance, data entry issues, and private provider reluctance |  |  |
| E Barroso (134) | 2024 | 2021.9-2021.12 | United States | Immigrants | Mixed-method study | 13/17 (76.47) | Patients did not feel sick, being absent from work, potential medication side effects, other medical issues were prioritized over TB care | 8/14 (57.14) |  |
| TB, tuberculosis; LTBI, latent tuberculosis infection; MDR-TB, multiple drug-resistant tuberculosis; HIV, Human Immunodeficiency Virus; AIDS, Acquired Immune Deficiency Syndrome; INH, isoniazid; RIF, rifampin; TPT, preventive treatment; TST, tuberculin skin test; IGRA, interferon gamma release assay. | | | | | | | | | |

**References**

1. Anibarro L, Casas S, Paz-Esquete J, Gonzalez L, Pena A, Guerra MR, Sande D, Calviño L, Santin M. Treatment completion in latent tuberculosis infection at specialist tuberculosis units in Spain. Int J Tuberc Lung Dis. 2010 Jun;14(6):701-7. eng. Epub 2010/05/22. Cited in: Pubmed; PMID 20487607.

2. Diaz A, Diez M, Bleda MJ, Aldamiz M, Camafort M, Camino X, Cepeda C, Costa A, Ferrero O, Geijo P, Iribarren JA, Moreno S, Moreno ME, Labarga P, Pinilla J, Portu J, Pulido F, Rosa C, Santamaría JM, Telenti M, Trapiella L, Trastoy M, Viciana P. Eligibility for and outcome of treatment of latent tuberculosis infection in a cohort of HIV-infected people in Spain. BMC Infect Dis. 2010 Sep 14;10:267. eng. Epub 2010/09/16. doi:10.1186/1471-2334-10-267. Cited in: Pubmed; PMID 20840743.

3. Harstad I, Heldal E, Steinshamn SL, Garåsen H, Winje BA, Jacobsen GW. Screening and treatment of latent tuberculosis in a cohort of asylum seekers in Norway. Scand J Public Health. 2010 May;38(3):275-82. eng. Epub 2009/11/17. doi:10.1177/1403494809353823. Cited in: Pubmed; PMID 19914972.

4. Horsburgh CR, Jr., Goldberg S, Bethel J, Chen S, Colson PW, Hirsch-Moverman Y, Hughes S, Shrestha-Kuwahara R, Sterling TR, Wall K, Weinfurter P. Latent TB infection treatment acceptance and completion in the United States and Canada. Chest. 2010 Feb;137(2):401-9. eng. Epub 2009/10/02. doi:10.1378/chest.09-0394. Cited in: Pubmed; PMID 19793865.

5. Li J, Munsiff SS, Tarantino T, Dorsinville M. Adherence to treatment of latent tuberculosis infection in a clinical population in New York City. Int J Infect Dis. 2010 Apr;14(4):e292-7. eng. Epub 2009/08/07. doi:10.1016/j.ijid.2009.05.007. Cited in: Pubmed; PMID 19656705.

6. Goswami ND, Gadkowski LB, Piedrahita C, Bissette D, Ahearn MA, Blain ML, Østbye T, Saukkonen J, Stout JE. Predictors of latent tuberculosis treatment initiation and completion at a U.S. public health clinic: a prospective cohort study. BMC Public Health. 2012 Jun 21;12:468. eng. Epub 2012/06/23. doi:10.1186/1471-2458-12-468. Cited in: Pubmed; PMID 22720842.

7. Young J, Edick T, Klee D, O'Connor ME. Successful treatment of pediatric latent tuberculosis infection in a community health center clinic. Pediatr Infect Dis J. 2012 Sep;31(9):e147-51. eng. Epub 2012/04/26. doi:10.1097/INF.0b013e318257f7c6. Cited in: Pubmed; PMID 22531235.

8. Colson PW, Hirsch-Moverman Y, Bethel J, Vempaty P, Salcedo K, Wall K, Miranda W, Collins S, Horsburgh CR. Acceptance of treatment for latent tuberculosis infection: prospective cohort study in the United States and Canada. Int J Tuberc Lung Dis. 2013 Apr;17(4):473-9. eng. Epub 2013/03/15. doi:10.5588/ijtld.12.0697. Cited in: Pubmed; PMID 23485381.

9. Kan B, Kalin M, Bruchfeld J. Completing treatment for latent tuberculosis: Patient background matters [Article]. International Journal of Tuberculosis and Lung Disease. 2013;17(5):597-602. doi:10.5588/ijtld.12.0692.

10. Moolphate S, Lawpoolsri S, Pungrassami P, Sanguanwongse N, Yamada N, Kaewkungwal J. Barriers to and motivations for the implementation of a treatment programme for latent tuberculosis infection using isoniazid for people living with HIV, in upper northern Thailand. Glob J Health Sci. 2013 Mar 25;5(4):60-70. eng. Epub 2013/06/20. doi:10.5539/gjhs.v5n4p60. Cited in: Pubmed; PMID 23777722.

11. Morano JP, Walton MR, Zelenev A, Bruce RD, Altice FL. Latent tuberculosis infection: screening and treatment in an urban setting [; Research Support, N.I.H., Extramural; Research Support, Non-U.S. Gov't]. Journal of community health. 2013 2013-Oct;38(5):941-50. doi:10.1007/s10900-013-9704-y. Cited in: Pubmed; PMID MEDLINE:23728822.

12. Pettit AC, Bethel J, Hirsch-Moverman Y, Colson PW, Sterling TR. Female sex and discontinuation of isoniazid due to adverse effects during the treatment of latent tuberculosis. J Infect. 2013 Nov;67(5):424-32. eng. Epub 2013/07/13. doi:10.1016/j.jinf.2013.07.015. Cited in: Pubmed; PMID 23845828.

13. Chang SH, Eitzman SR, Nahid P, Finelli ML. Factors associated with failure to complete isoniazid therapy for latent tuberculosis infection in children and adolescents. J Infect Public Health. 2014 Mar-Apr;7(2):145-52. eng. Epub 2013/12/24. doi:10.1016/j.jiph.2013.11.001. Cited in: Pubmed; PMID 24361084.

14. Fiske CT, Yan FX, Hirsch-Moverman Y, Sterling TR, Reichler MR. Risk factors for treatment default in close contacts with latent tuberculous infection. Int J Tuberc Lung Dis. 2014 Apr;18(4):421-7. eng. Epub 2014/03/29. doi:10.5588/ijtld.13.0688. Cited in: Pubmed; PMID 24670696.

15. Malejczyk K, Gratrix J, Beckon A, Moreau D, Williams G, Kunimoto D, Ahmed R. Factors associated with noncompletion of latent tuberculosis infection treatment in an inner-city population in Edmonton, Alberta. Can J Infect Dis Med Microbiol. 2014 Sep;25(5):281-4. eng. Epub 2014/11/06. doi:10.1155/2014/349138. Cited in: Pubmed; PMID 25371692.

16. Sidhu A, Verma G, Humar A, Kumar D. Outcome of latent tuberculosis infection in solid organ transplant recipients over a 10-year period. Transplantation. 2014 Sep 27;98(6):671-5. eng. Epub 2014/05/16. doi:10.1097/tp.0000000000000133. Cited in: Pubmed; PMID 24825525.

17. Aquino DS, Moura LC, Maruza M, Silva AP, Ximenes RA, Lacerda HR, Miranda Filho De, Albuquerque Me. Factors associated with treatment for latent tuberculosis in persons living with HIV/AIDS [Article]. Cadernos de saúde pública. 2015;31(12):2505-2513. English. doi:10.1590/0102-311X00154614.

18. Bishara H, Ore L, Vinitsky O, Bshara H, Armaly N, Weiler-Ravell D. Cost of nurse-managed latent tuberculous infection treatment among hard-to-reach immigrants in Israel. Int J Tuberc Lung Dis. 2015 Jul;19(7):799-804. eng. Epub 2015/06/10. doi:10.5588/ijtld.14.0674. Cited in: Pubmed; PMID 26056104.

19. Hirsch-Moverman Y, Shrestha-Kuwahara R, Bethel J, Blumberg HM, Venkatappa TK, Horsburgh CR, Colson PW. Latent tuberculous infection in the United States and Canada: who completes treatment and why? Int J Tuberc Lung Dis. 2015 Jan;19(1):31-8. eng. Epub 2014/12/19. doi:10.5588/ijtld.14.0373. Cited in: Pubmed; PMID 25519787.

20. Nuzzo JB, Golub JE, Chaulk P, Shah M. Analysis of latent tuberculosis infection treatment adherence among refugees and other patient groups referred to the Baltimore City Health Department TB clinic, February 2009-March 2011. J Immigr Minor Health. 2015 Feb;17(1):56-65. eng. Epub 2013/08/03. doi:10.1007/s10903-013-9882-9. Cited in: Pubmed; PMID 23907316.

21. Mendonça AM, Kritski AL, Land MG, Sant'Anna CC. Abandonment of Treatment for Latent Tuberculosis Infection and Socioeconomic Factors in Children and Adolescents: Rio De Janeiro, Brazil. PLoS One. 2016;11(5):e0154843. eng. Epub 2016/05/07. doi:10.1371/journal.pone.0154843. Cited in: Pubmed; PMID 27149514.

22. Okoli EI, Roets L. Health system challenges: An obstacle to the success of isoniazid preventive therapy. S Afr Med J. 2016 Nov 2;106(11):1079-1081. eng. Epub 2016/11/16. doi:10.7196/SAMJ.2016.v106i11.10741. Cited in: Pubmed; PMID 27842624.

23. Pathak V, Harrington Z, Dobler CC. Attitudes towards preventive tuberculosis treatment among hospital staff. PeerJ. 2016;4:e1738. eng. Epub 2016/03/12. doi:10.7717/peerj.1738. Cited in: Pubmed; PMID 26966667.

24. Rennert-May E, Hansen E, Zadeh T, Krinke V, Houston S, Cooper R. A Step toward Tuberculosis Elimination in a Low-Incidence Country: Successful Diagnosis and Treatment of Latent Tuberculosis Infection in a Refugee Clinic. Can Respir J. 2016;2016:7980869. eng. Epub 2016/07/23. doi:10.1155/2016/7980869. Cited in: Pubmed; PMID 27445565.

25. Walters JK, Sullivan AD. Impact of Routine Quantiferon Testing on Latent Tuberculosis Diagnosis and Treatment in Refugees in Multnomah County, Oregon, November 2009-October 2012. J Immigr Minor Health. 2016 Apr;18(2):292-300. eng. Epub 2015/03/19. doi:10.1007/s10903-015-0187-z. Cited in: Pubmed; PMID 25784140.

26. Ayele AA, Asrade Atnafie S, Balcha DD, Weredekal AT, Woldegiorgis BA, Wotte MM, Gebresillasie BM. Self-reported adherence and associated factors to isoniazid preventive therapy for latent tuberculosis among people living with HIV/AIDS at health centers in Gondar town, North West Ethiopia. Patient Prefer Adherence. 2017;11:743-749. eng. Epub 2017/04/25. doi:10.2147/ppa.S131314. Cited in: Pubmed; PMID 28435232.

27. Eastment MC, McClintock AH, McKinney CM, Narita M, Molnar A. Factors That Influence Treatment Completion for Latent Tuberculosis Infection. J Am Board Fam Med. 2017 Jul-Aug;30(4):520-527. eng. Epub 2017/07/20. doi:10.3122/jabfm.2017.04.170070. Cited in: Pubmed; PMID 28720633.

28. Kawatsu L, Uchimura K, Ohkado A. Trend and treatment status of latent tuberculosis infection patients in Japan - Analysis of Japan TB Surveillance data. PLoS One. 2017;12(11):e0186588. eng. Epub 2017/11/02. doi:10.1371/journal.pone.0186588. Cited in: Pubmed; PMID 29091917.

29. Sandul AL, Nwana N, Holcombe JM, Lobato MN, Marks S, Webb R, Wang SH, Stewart B, Griffin P, Hunt G, Shah N, Marco A, Patil N, Mukasa L, Moro RN, Jereb J, Mase S, Chorba T, Bamrah-Morris S, Ho CS. High Rate of Treatment Completion in Program Settings with 12-Dose Weekly Isoniazid and Rifapentine for Latent Mycobacterium tuberculosis Infection [Article]. Clinical Infectious Diseases. 2017;65(7):1085-1093. English. doi:10.1093/cid/cix505.

30. Bonvicini F, Cilloni S, Fornaciari R, Casoni C, Marchesi C, Greci M, Monici L, Nicolini F, Vinceti M. Compliance with tuberculosis screening in irregular immigrants [Article]. International Journal of Environmental Research and Public Health. 2019;16(1). English. doi:10.3390/ijerph16010028.

31. Huang H, Yuan G, Du Y, Cai X, Liu J, Hu C, Liang B, Hu G, Tang X, Zhou Y. Effects of preventive therapy for latent tuberculosis infection and factors associated with treatment abandonment: a cross-sectional study. J Thorac Dis. 2018 Jul;10(7):4377-4386. eng. Epub 2018/09/04. doi:10.21037/jtd.2018.06.138. Cited in: Pubmed; PMID 30174886.

32. Loutet MG, Burman M, Jayasekera N, Trathen D, Dart S, Kunst H, Zenner D. National roll-out of latent tuberculosis testing and treatment for new migrants in England: A retrospective evaluation in a high-incidence area [Article]. European Respiratory Journal. 2018;51(1). English. doi:10.1183/13993003.01226-2017.

33. Macaraig MM, Jalees M, Lam C, Burzynski J. Improved treatment completion with shorter treatment regimens for latent tuberculous infection. Int J Tuberc Lung Dis. 2018 Nov 1;22(11):1344-1349. eng. Epub 2018/10/26. doi:10.5588/ijtld.18.0035. Cited in: Pubmed; PMID 30355415.

34. Olsson O, Winqvist N, Olsson M, Olsson P, Björkman P. High rate of latent tuberculosis treatment completion in immigrants seeking asylum in Sweden. Infect Dis (Lond). 2018 Sep;50(9):678-686. eng. Epub 2018/04/06. doi:10.1080/23744235.2018.1459046. Cited in: Pubmed; PMID 29620426.

35. Schein YL, Madebo T, Andersen HE, Arnesen TM, Dyrhol-Riise AM, Tveiten H, White RA, Winje BA. Treatment completion for latent tuberculosis infection in Norway: a prospective cohort study. BMC Infect Dis. 2018 Nov 19;18(1):587. Epub 2018/11/21. doi:10.1186/s12879-018-3468-z. Cited in: Pubmed; PMID 30453946.

36. Scolarici M, Dekitani K, Chen L, Sokol-Anderson M, Hoft DF, Chatterjee S. A scoring strategy for progression risk and rates of treatment completion in subjects with latent tuberculosis. PLoS One. 2018;13(11):e0207582. eng. Epub 2018/11/16. doi:10.1371/journal.pone.0207582. Cited in: Pubmed; PMID 30440033.

37. Stockbridge EL, Miller TL, Carlson EK, Ho C. Predictors of latent tuberculosis infection treatment completion in the US private sector: an analysis of administrative claims data [Article]. BMC public health. 2018;18(1):662. English. doi:10.1186/s12889-018-5578-3.

38. Graves SK, Augusto O, Viegas SO, Lederer P, David C, Lee K, Hassane A, Cossa A, Amade S, Peleve S, Zindoga P, Massawo L, Torriani FJ, Nunes EA. Tuberculosis infection risk, preventive therapy care cascade and incidence of tuberculosis disease in healthcare workers at Maputo Central Hospital. BMC Infect Dis. 2019 Apr 25;19(1):346. eng. Epub 2019/04/27. doi:10.1186/s12879-019-3966-7. Cited in: Pubmed; PMID 31023260.

39. Lal A, Al Hammadi A, Rapose A. Latent Tuberculosis Infection: Treatment Initiation and Completion Rates in Persons Seeking Immigration and Health Care Workers. Am J Med. 2019 Nov;132(11):1353-1355. eng. Epub 2019/06/04. doi:10.1016/j.amjmed.2019.04.036. Cited in: Pubmed; PMID 31153865.

40. Lee H, Koo GW, Min JH, Park TS, Park DW, Moon JY, Kim SH, Kim TH, Yoon HJ, Sohn JW. Factors associated with non-initiation of latent tuberculosis treatment among healthcare workers with a positive interferon-gamma releasing assay. Sci Rep. 2019 Jan 11;9(1):61. eng. Epub 2019/01/13. doi:10.1038/s41598-018-37319-7. Cited in: Pubmed; PMID 30635600.

41. Pease C, Zwerling A, Mallick R, Patterson M, Demaio P, Finn S, Allen J, Van Dyk D, Alvarez GG. The latent tuberculosis infection cascade of care in Iqaluit, Nunavut, 2012-2016. BMC Infect Dis. 2019 Oct 24;19(1):890. eng. Epub 2019/10/28. doi:10.1186/s12879-019-4557-3. Cited in: Pubmed; PMID 31651260.

42. Plourde PJ, Basham CA, Derksen S, Schultz J, McCulloch S, Larcombe L, Kinew KA, Lix LM. Latent tuberculosis treatment completion rates from prescription drug administrative data. Can J Public Health. 2019 Dec;110(6):705-713. eng. Epub 2019/07/13. doi:10.17269/s41997-019-00240-1. Cited in: Pubmed; PMID 31297736.

43. Séraphin MN, Hsu H, Chapman HJ, de Andrade Bezerra JL, Johnston L, Yang Y, Lauzardo M. Timing of treatment interruption among latently infected tuberculosis cases treated with a nine-month course of daily isoniazid: findings from a time to event analysis. BMC Public Health. 2019 Sep 3;19(1):1214. eng. Epub 2019/09/05. doi:10.1186/s12889-019-7524-4. Cited in: Pubmed; PMID 31481046.

44. Villa S, Ferrarese M, Sotgiu G, Castellotti PF, Saderi L, Grecchi C, Saporiti M, Raviglione M, Codecasa LR. Latent Tuberculosis Infection Treatment Completion while Shifting Prescription from Isoniazid-Only to Rifampicin-Containing Regimens: A Two-Decade Experience in Milan, Italy. J Clin Med. 2019 Dec 31;9(1). eng. Epub 2020/01/08. doi:10.3390/jcm9010101. Cited in: Pubmed; PMID 31906078.

45. Araújo NCN, Cruz CMS, Arriaga MB, Cubillos-Angulo JM, Rocha MS, Silveira-Mattos PS, Matos GM, Marques IMB, Espirito Santo ICP, Almeida LL, Andrade CM, Souza LA, Netto EM, Andrade BB. Determinants of losses in the latent tuberculosis cascade of care in Brazil: A retrospective cohort study [Article]. International Journal of Infectious Diseases. 2020;93:277-283. English. doi:10.1016/j.ijid.2020.02.015.

46. Chung SJ, Lee H, Koo GW, Min JH, Yeo Y, Park DW, Park TS, Moon JY, Kim SH, Kim TH, Sohn JW, Yoon HJ. Adherence to nine-month isoniazid for latent tuberculosis infection in healthcare workers: a prospective study in a tertiary hospital. Sci Rep. 2020 Apr 15;10(1):6462. eng. Epub 2020/04/17. doi:10.1038/s41598-020-63156-8. Cited in: Pubmed; PMID 32296096.

47. Feng JY, Huang WC, Lin SM, Wang TY, Lee SS, Shu CC, Pan SW, Chen CY, Lin CB, Wei YF, Tung CL, Li CP, Su WJ. Safety and treatment completion of latent tuberculosis infection treatment in the elderly population-A prospective observational study in Taiwan. Int J Infect Dis. 2020 Jul;96:550-557. Epub 2020/05/21. doi:10.1016/j.ijid.2020.05.009. Cited in: Pubmed; PMID 32434083.

48. Legese H, Degefa H, Gebrewahd A, Gebremedhin H. Utilization of isoniazid prophylaxis therapy and its associated factors among HIV positive clients taking antiretroviral therapy at Fre Semaetat primary hospital, Hawzien districts, Tigrai, Northern Ethiopia. Trop Dis Travel Med Vaccines. 2020;6:11. eng. Epub 2020/06/23. doi:10.1186/s40794-020-00106-2. Cited in: Pubmed; PMID 32566242.

49. Manful A, Waller L, Katz B, Cummins J, Warkentin J, Reagon B, Shaw-Kaikai J, Zhu Y, van der Heijden YF. Gaps in the care cascade for screening and treatment of refugees with tuberculosis infection in Middle Tennessee: a retrospective cohort study. BMC Infect Dis. 2020 Aug 10;20(1):592. eng. Epub 2020/08/12. doi:10.1186/s12879-020-05311-0. Cited in: Pubmed; PMID 32778060.

50. Nguyen Truax F, Morisky D, Low J, Carson M, Girma H, Nyamathi A. Non-completion of latent tuberculosis infection treatment among Vietnamese immigrants in Southern California: A retrospective study. Public Health Nurs. 2020 Nov;37(6):846-853. eng. Epub 2020/09/09. doi:10.1111/phn.12798. Cited in: Pubmed; PMID 32896018.

51. Sensalire S, Karungi Karamagi Nkolo E, Nabwire J, Lawino A, Kiragga D, Muhire M, Kadama H, Katureebe C, Namuwenge P, Musinguzi J, Calnan J, Seyoum D. A prospective cohort study of outcomes for isoniazid prevention therapy: a nested study from a national QI collaborative in Uganda. AIDS Res Ther. 2020 May 27;17(1):28. eng. Epub 2020/05/29. doi:10.1186/s12981-020-00285-0. Cited in: Pubmed; PMID 32460788.

52. Sentís A, Vasconcelos P, Machado RS, Caylà JA, Guxens M, Peixoto V, Duarte R, Carvalho I, Carvalho C. Failure to complete treatment for latent tuberculosis infection in Portugal, 2013-2017: geographic-, sociodemographic-, and medical-associated factors. Eur J Clin Microbiol Infect Dis. 2020 Apr;39(4):647-656. eng. Epub 2019/12/05. doi:10.1007/s10096-019-03765-y. Cited in: Pubmed; PMID 31797155.

53. Sugishita Y, Goto C, Sakamoto T, Sugawara T, Ohkusa Y. Risk factors affecting the failure to complete treatment for patients with latent tuberculosis infection in Tokyo, Japan [Article]. Journal of Infection and Chemotherapy. 2020;26(11):1129-1133. English. doi:10.1016/j.jiac.2020.05.007.

54. Swift MD, Molella RG, Vaughn AIS, Breeher LE, Newcomb RD, Abdellatif S, Hassan Murad M. Determinants of latent tuberculosis treatment acceptance and completion in healthcare personnel [Article]. Clinical Infectious Diseases. 2020;71(2):284-290. doi:10.1093/cid/ciz817.

55. Bar-Meir M, Pariente G, Romem A, Wiener-Well Y. Identifying factors affecting latent tuberculosis treatment acceptance among healthcare workers: A retrospective analysis in a tertiary care centre [Article]. BMJ Open. 2021;11(9). doi:10.1136/bmjopen-2020-047444.

56. Gatechompol S, Harnpariphan W, Supanan R, Suwanpimolkul G, Sophonphan J, Ubolyam S, Kerr SJ, Avihingsanon A, Kawkitinarong K. Prevalence of latent tuberculosis infection and feasibility of TB preventive therapy among Thai prisoners: a cross-sectional study. BMC Public Health. 2021 Jun 24;21(1):1206. eng. Epub 2021/06/25. doi:10.1186/s12889-021-11271-0. Cited in: Pubmed; PMID 34162348.

57. Iqbal SA, Isenhour CJ, Mazurek G, Langer AJ, Chang MH, Truman BI. Factors Associated With Latent Tuberculosis Infection Treatment Failure Among Patients With Commercial Health Insurance-United States, 2005-2016. J Public Health Manag Pract. 2021 Jul-Aug 01;27(4):E151-e161. eng. Epub 2019/11/07. doi:10.1097/phh.0000000000001077. Cited in: Pubmed; PMID 31688742.

58. Sullivan K, Pease C, Zwerling A, Mallick R, Van Dyk D, Mulpuru S, Allen C, Alsdurf H, Alvarez GG. Seven-year retrospective study understanding the latent TB infection treatment cascade of care among adults in a low incidence country. BMC Public Health. 2021 May 21;21(1):964. eng. Epub 2021/05/23. doi:10.1186/s12889-021-10733-9. Cited in: Pubmed; PMID 34020616.

59. Van Ginderdeuren E, Bassett J, Hanrahan CF, Mutunga L, Van Rie A. Gaps in the tuberculosis preventive therapy care cascade in children in contact with TB [Article]. Paediatrics and International Child Health. 2021;41(4):237-246. English. doi:10.1080/20469047.2021.1971360.

60. Wu CH, Su HA, Chou CA, Liu JW, Lee CT, Dai LH, Yang CC. An observational study on prevalence of latent tuberculosis infection and outcome of 3HP treatment in patients under hemodialysis in Taiwan. J Formos Med Assoc. 2021 Jun;120(6):1350-1360. eng. Epub 2021/06/03. doi:10.1016/j.jfma.2020.10.008. Cited in: Pubmed; PMID 34074499.

61. Almugti HS, Alfaleh HM, Alshehri TM, Mokili KQ, Al Qahtani AM, Al Qahtani HS, Alsayed MZ, Al Asmari MA, Al Asiri MM, Al Amri MA, Al Fadhil AF, Al Qahtani BA, Al Bakrah ES, Shaikh HA, Al Shiq MG, Al Shaik YA. Management of Latent Tuberculosis Infection in Saudi Arabia: Knowledge and Perceptions Among Healthcare Workers. Cureus. 2022 Sep;14(9):e29134. eng. Epub 2022/10/20. doi:10.7759/cureus.29134. Cited in: Pubmed; PMID 36259033.

62. Chiu TF, Yen MY, Shie YH, Huang HL, Chen CC, Yen YF. Determinants of latent tuberculosis infection and treatment interruption in long-term care facilities: A retrospective cohort study in Taiwan [Article]. Journal of Microbiology, Immunology and Infection. 2022;55(6):1310-1317. doi:10.1016/j.jmii.2021.09.013.

63. Fa L, Xu C, Cheng J, Zhang H. Acceptability of Tuberculosis Preventive Treatment Strategies Among Healthcare Workers Using an Online Survey - China, 2021. China CDC Wkly. 2022 Mar 18;4(11):211-215. Epub 2022/04/19. doi:10.46234/ccdcw2022.050. Cited in: Pubmed; PMID 35433078.

64. Holmes RH, Sun S, Kazi S, Ranganathan S, Tosif S, Graham SM, Graham HR. Management of tuberculosis infection in Victorian children: A retrospective clinical audit of factors affecting treatment completion. PLoS One. 2022;17(10):e0275789. eng. Epub 2022/10/14. doi:10.1371/journal.pone.0275789. Cited in: Pubmed; PMID 36227875.

65. Holzman SB, Perry A, Saleeb P, Pyan A, Keh C, Salcedo K, Narita M, Ahmed A, Miller TL, Pettit AC, Khurana R, Whipple M, Katz D, Largen A, Krueger A, Shah M. Evaluation of the Latent Tuberculosis Care Cascade among Public Health Clinics in the United States [Article]. Clinical Infectious Diseases. 2022;75(10):1792-1799. English. doi:10.1093/cid/ciac248.

66. Lin KY, Yang CJ, Sun HY, Lee YT, Liou BH, Hii IM, Chen TC, Huang SH, Lee CY, Tsai CS, Lin CY, Liu CE, Chang HY, Cheng CY, Lu PL, Hung CC. Care cascade of tuberculosis infection treatment for people living with HIV in the era of antiretroviral therapy scale-up. Sci Rep. 2022 Sep 27;12(1):16136. eng. Epub 2022/09/28. doi:10.1038/s41598-022-20394-2. Cited in: Pubmed; PMID 36167744.

67. Min J, Kim HW, Choi JY, Shin AY, Kang JY, Lee Y, Myong JP, Jeong H, Bae S, Koo HK, Lee SS, Park JS, Yim HW, Kim JS. Latent Tuberculosis Cascade of Care Among Healthcare Workers: A Nationwide Cohort Analysis in Korea Between 2017 and 2018. J Korean Med Sci. 2022 May 23;37(20):e164. eng. Epub 2022/05/25. doi:10.3346/jkms.2022.37.e164. Cited in: Pubmed; PMID 35607742.

68. Min J, Kim HW, Stagg HR, Rangaka MX, Lipman M, Abubakar I, Lee Y, Myong JP, Jeong H, Bae S, Shin AY, Kang JY, Lee SS, Park JS, Yim HW, Kim JS. The cascade of care for latent tuberculosis infection in congregate settings: A national cohort analysis, Korea, 2017-2018. Front Med (Lausanne). 2022;9:927579. eng. Epub 2022/10/04. doi:10.3389/fmed.2022.927579. Cited in: Pubmed; PMID 36186763.

69. Runels T, Ragan EJ, Ventura AS, Winter MR, White LF, Horsburgh CR, Samet JH, Saitz R, Jacobson KR. Testing and treatment for latent tuberculosis infection in people living with HIV and substance dependence: A prospective cohort study [Article]. BMJ Open. 2022;12(3). doi:10.1136/bmjopen-2021-058751.

70. Sharma N, Basu S, Khanna A, Sharma P, Chandra S. The intention to receive tuberculosis preventive therapy in adult household contacts of pulmonary TB patients in Delhi, India [Article]. Journal of Infection in Developing Countries. 2022;16(2):298-304. English. doi:10.3855/jidc.14910.

71. Sobral L, Arriaga MB, Souza AB, Araújo-Pereira M, Barreto-Duarte B, Sales C, Rocha MS, Benjamin A, Moreira ASR, de Oliveira JG, Carvalho AC, Spener-Gomes R, Figueiredo MC, Cavalcante S, Durovni B, Lapa ESJR, Kritski AL, Rolla VC, Sterling TR, Cordeiro-Santos M, Andrade BB. Determinants of losses in the tuberculosis infection cascade of care among children and adolescent contacts of pulmonary tuberculosis cases: A Brazilian multi-centre longitudinal study. Lancet Reg Health Am. 2022 Nov;15. eng. Epub 2022/11/29. doi:10.1016/j.lana.2022.100358. Cited in: Pubmed; PMID 36438860.

72. Souza AB, Arriaga MB, Amorim G, Araújo-Pereira M, Nogueira BMF, Queiroz ATL, Figueiredo MC, Rocha MS, Benjamin A, Moreira ASR, Oliveira JG, Rolla V, Durovni B, Lapa ESJR, Kritski AL, Cavalcante S, Sterling T, Andrade BB, Cordeiro-Santos M. Determinants of losses in the latent tuberculosis infection cascade of care in Brazil. BMJ Glob Health. 2021 Sep;6(9). eng. Epub 2021/09/15. doi:10.1136/bmjgh-2021-005969. Cited in: Pubmed; PMID 34518204.

73. Adepoju VA, Adelekan A, Agbaje A, Quaitey F, Ademola-Kay T, Udoekpo AU, Sokoya OD. Completion of 6-mo isoniazid preventive treatment among eligible under six children: A cross-sectional study, Lagos, Nigeria. World J Clin Cases. 2023 Jan 6;11(1):104-115. eng. Epub 2023/01/24. doi:10.12998/wjcc.v11.i1.104. Cited in: Pubmed; PMID 36687175.

74. Bruxvoort KJ, Skarbinski J, Fischer H, Li Z, Eaton A, Qian L, Spence B, Wei R, Rieg G, Shaw S, Tartof SY. Latent Tuberculosis Infection Treatment Practices in Two Large Integrated Health Systems in California, 2009-2018. Open Forum Infect Dis. 2023 May;10(5):ofad219. eng. Epub 2023/06/02. doi:10.1093/ofid/ofad219. Cited in: Pubmed; PMID 37265669.

75. Cochran J, Tibbs A, Haptu HH, Paradise RK, Bernardo J, Tierney DB. Scaling Up Latent Tuberculosis Infection Testing and Treatment for Non-US Born Patients in a Federally Qualified Community Health Center [Article]. Journal of immigrant and minority health. 2023;25(6):1482-1487. English. doi:10.1007/s10903-023-01514-0.

76. Levine S, Fraulino D, Krupka P, Velamakanni S. Latent tuberculosis infection in the outpatient general medicine clinic: Efficacy of a nurse-run electronic directly observed treatment program. Prev Med Rep. 2023 Oct;35:102321. eng. Epub 2023/07/31. doi:10.1016/j.pmedr.2023.102321. Cited in: Pubmed; PMID 37519447.

77. Ortiz Laza N, Lopez Aranaga I, Toral Andres J, Toja Uriarte B, Santos Zorrozua B, Altube Urrengoechea L, Garros Garay J, Tabernero Huguet E. Latent tuberculosis infection treatment completion in Biscay: differences between regimens and monitoring approaches. Front Med (Lausanne). 2023;10:1265057. eng. Epub 2023/11/29. doi:10.3389/fmed.2023.1265057. Cited in: Pubmed; PMID 38020141.

78. Stantliff TM, Houshel L, Goswami R, Millow S, Cook G, Knapmeyer R, Easton C, Stryker SD, Williams KM, Walter M, Mooney J, Huaman MA. The latent tuberculosis infection cascade of care during the COVID-19 pandemic response in a Mid-Sized US city. J Clin Tuberc Other Mycobact Dis. 2023 May;31:100367. eng. Epub 2023/04/11. doi:10.1016/j.jctube.2023.100367. Cited in: Pubmed; PMID 37034439.

79. Vo LNQ, Nguyen VN, Nguyen NTT, Dong TTT, Codlin A, Forse R, Truong HT, Nguyen HB, Dang HTM, Truong VV, Nguyen LH, Mac TH, Le PT, Tran KT, Ndunda N, Caws M, Creswell J. Optimising diagnosis and treatment of tuberculosis infection in community and primary care settings in two urban provinces of Viet Nam: a cohort study. BMJ Open. 2023 Feb 9;13(2):e071537. eng. Epub 2023/02/10. doi:10.1136/bmjopen-2022-071537. Cited in: Pubmed; PMID 36759036.

80. Yang H, Lee JS, Kim Y. Healthcare workers' acceptance of and adherence to latent tuberculosis treatment. Occup Med (Lond). 2023 May 18;73(4):186-192. eng. Epub 2023/03/28. doi:10.1093/occmed/kqad036. Cited in: Pubmed; PMID 36972514.

81. Yuan Y, Jin J, Bi X, Geng H, Li S, Zhou C. Factors associated with refusal of preventive therapy after initial willingness to accept treatment among college students with latent tuberculosis infection in Shandong, China. BMC Infect Dis. 2023 Jan 20;23(1):38. eng. Epub 2023/01/21. doi:10.1186/s12879-023-08005-5. Cited in: Pubmed; PMID 36670356.

82. An Y, Khun KE. Factors associated with incomplete tuberculosis preventive treatment: a retrospective analysis of six-years programmatic data in Cambodia. Sci Rep. 2024 Aug 9;14(1):18458. eng. Epub 2024/08/10. doi:10.1038/s41598-024-67845-6. Cited in: Pubmed; PMID 39122738.

83. Bidashimwa D, Ditekemena JD, Sigwadhi LN, Nkuta LM, Engetele E, Kilundu A, Chabikuli ON, Nachega JB. Completion of isoniazid preventive therapy for latent tuberculosis infection among children and adolescents compared to adults living with HIV in Kinshasa, Democratic Republic of the Congo [Article]. Tropical Medicine and International Health. 2024;29(2):88-95. doi:10.1111/tmi.13952.

84. Chen H, Zhang H, Cheng J, Sun D, Wang Q, Wu C, Liu Y, Xia Y, Xu C, Zhang C. Adherence to Preventive Treatment for Latent Tuberculosis Infection in Close Contacts of Pulmonary Tuberculosis Patients: A Cluster-Randomized Controlled Trial in China. International journal of infectious diseases : IJID : official publication of the International Society for Infectious Diseases. 2024 2024-Jul-27:107196-107196. doi:10.1016/j.ijid.2024.107196. Cited in: Pubmed; PMID MEDLINE:39074738.

85. Chiu CY, Mahmood M, Brumble LM, Vikram HR, Theel ES, Beam E. The Cascade of Care in Management of Solid Organ Transplant Candidates With Latent Tuberculosis Infection. Transplant Direct. 2024 Jul;10(7):e1672. eng. Epub 2024/06/24. doi:10.1097/txd.0000000000001672. Cited in: Pubmed; PMID 38911278.

86. Gebreegziabher SB, Ashuro AA, Kumssa TH, Teferi MY, Alemayue EA, Datiko DG, Yimer SA, Shagre MB. Tuberculosis preventive treatment uptake among people living with HIV during COVID-19 period in Addis Ababa, Ethiopia: a retrospective data review. BMC Infect Dis. 2024 May 17;24(1):499. eng. Epub 2024/05/18. doi:10.1186/s12879-024-09403-z. Cited in: Pubmed; PMID 38760665.

87. Gray A, Surey J, Veitch M, Menezes D, Gibbons J, Leonard M, Sultan B, Esmail H, Story A. Diagnosis and management of tuberculosis infection in inclusion health populations in London. BMC Infect Dis. 2024 Feb 23;24(1):252. eng. Epub 2024/02/24. doi:10.1186/s12879-024-09132-3. Cited in: Pubmed; PMID 38395793.

88. Kim Y, Bae KS, Choi UY, Han SB, Kim JH. Current Status of Latent Tuberculosis Infection Treatment Among Pediatric Patients in Korea: Prescription and Treatment Completion [Article]. Journal of Korean medical science. 2024;39(7):e64. English. doi:10.3346/jkms.2024.39.e64.

89. Plans-Rubió P, Godoy S, Toledo D, Domínguez A, Caylà J, Parron I, Millet JP, Godoy P. Factors Associated with Non-Adherence to Tuberculosis Preventive Treatment among Adult Contacts of Pulmonary Tuberculosis Cases with Latent Tuberculosis Infection in Catalonia, Spain, in 2019-2021. Trop Med Infect Dis. 2024 Feb 27;9(3). eng. Epub 2024/03/27. doi:10.3390/tropicalmed9030054. Cited in: Pubmed; PMID 38535878.

90. Tegegnework AZ, Aemiro MT, Bilchut AH, Mekuria AD, Yehualashet SS. Completion of tuberculosis preventive therapy and associated factors among clients on antiretroviral therapy at Debre Berhan town health facilities, North Shoa Zone, Ethiopia [Article]. AIDS Research and Therapy. 2024;21(1). English. doi:10.1186/s12981-024-00629-0.

91. Yuan Y, Jin J, Bi X, Geng H, Li S, Zhou C. Gender-Specific Association Between Perceived Stigma Toward Tuberculosis and Acceptance of Preventive Treatment Among College Students With Latent Tuberculosis Infection: Cross-Sectional Analysis. JMIR Public Health Surveill. 2023 Jun 14;9:e43972. Epub 2023/06/14. doi:10.2196/43972. Cited in: Pubmed; PMID 37314847.

92. Zakaria WMZ, Mansor Z. Intention to receive Latent Tuberculosis Infection (LTBI) treatment and its associated factors among healthcare workers in a Malaysian teaching university hospital. PLoS One. 2024;19(7):e0307199. eng. Epub 2024/07/18. doi:10.1371/journal.pone.0307199. Cited in: Pubmed; PMID 39024265.

93. Campbell JI, Lavache D, Garing A, Sabharwal V, Haberer JE, Dubois M, Jenkins HE, Brooks MB, Joseph NT, Kissler K, Horsburgh CR, Jacobson KR. Evaluation of the Tuberculosis Infection Care Cascade Among Pregnant Individuals in a Low-Tuberculosis-Burden Setting. Open Forum Infect Dis. 2024 Sep;11(9):ofae494. Epub 2024/09/06. doi:10.1093/ofid/ofae494. Cited in: Pubmed; PMID 39238842.

94. Asare-Baah M, Salmon-Trejo LAT, Venkatappa T, Garfein RS, Aiona K, Haas M, Seraphin MN. Factors Associated With the Discontinuation of Two Short-Course Tuberculosis Preventive Therapies in Programmatic Settings in the United States. Open Forum Infect Dis. 2024 Jun;11(6):ofae313. Epub 2024/06/25. doi:10.1093/ofid/ofae313. Cited in: Pubmed; PMID 38915338.

95. Colson PW, Franks J, Sondengam R, Hirsch-Moverman Y, El-Sadr W. Tuberculosis knowledge, attitudes, and beliefs in foreign-born and US-born patients with latent tuberculosis infection. J Immigr Minor Health. 2010 Dec;12(6):859-66. eng. Epub 2010/03/20. doi:10.1007/s10903-010-9338-4. Cited in: Pubmed; PMID 20237847.

96. Hill L, Blumberg E, Sipan C, Schmitz K, West J, Kelley N, Hovell M. Multi-level barriers to LTBI treatment: a research note. J Immigr Minor Health. 2010 Aug;12(4):544-50. eng. Epub 2008/12/17. doi:10.1007/s10903-008-9216-5. Cited in: Pubmed; PMID 19085104.

97. Wieland ML, Weis JA, Yawn BP, Sullivan SM, Millington KL, Smith CM, Bertram S, Nigon JA, Sia IG. Perceptions of tuberculosis among immigrants and refugees at an adult education center: a community-based participatory research approach. J Immigr Minor Health. 2012 Feb;14(1):14-22. eng. Epub 2010/09/21. doi:10.1007/s10903-010-9391-z. Cited in: Pubmed; PMID 20853177.

98. Babu K, Philips M, Subbakrishna DK. Perspectives of Quantiferon TB Gold test among Indian practitioners: a survey. J Ophthalmic Inflamm Infect. 2013 Jan 11;3(1):9. eng. Epub 2013/03/22. doi:10.1186/1869-5760-3-9. Cited in: Pubmed; PMID 23514565.

99. Jansen-Aaldring N, van de Berg S, van den Hof S. Patient support during treatment for active tuberculosis and for latent tuberculosis infection: Policies and practices in European low-incidence countries. J Adv Nurs. 2018 Dec;74(12):2755-2765. Epub 2018/07/03. doi:10.1111/jan.13784. Cited in: Pubmed; PMID 29964334.

100. Dobler CC, Bosnic-Anticevich S, Armour CL. Physicians' perspectives on communication and decision making in clinical encounters for treatment of latent tuberculosis infection. ERJ open research. 2018 2018-Jan;4(1). doi:10.1183/23120541.00146-2017. Cited in: Pubmed; PMID MEDLINE:29577042.

101. Berrocal-Almanza LC, Botticello J, Piotrowski H, Karnani N, Kon OM, Lalvani A, Zenner D. Engaging with civil society to improve access to LTBI screening for new migrants in England: a qualitative study. Int J Tuberc Lung Dis. 2019 May 1;23(5):563-570. eng. Epub 2019/05/18. doi:10.5588/ijtld.18.0230. Cited in: Pubmed; PMID 31097064.

102. Milinkovic DA, Birch S, Scott F, Newbold KB, Hopkins J, Saffie M, Essue BM. Low prioritization of latent tuberculosis infection-A systemic barrier to tuberculosis control: A qualitative study in Ontario, Canada. Int J Health Plann Manage. 2019 Jan;34(1):384-395. eng. Epub 2018/11/08. doi:10.1002/hpm.2670. Cited in: Pubmed; PMID 30402949.

103. Degeling C, Carter SM, Dale K, Singh K, Watts K, Hall J, Denholm J. Perspectives of Vietnamese, Sudanese and South Sudanese immigrants on targeting migrant communities for latent tuberculosis screening and treatment in low-incidence settings: A report on two Victorian community panels. Health Expect. 2020 Dec;23(6):1431-1440. eng. Epub 2020/09/13. doi:10.1111/hex.13121. Cited in: Pubmed; PMID 32918523.

104. Barss L, Obeng J, Fregonese F, Oxlade O, Adomako B, Afriyie AO, Frimpong ED, Winters N, Valiquette C, Menzies D. Solutions to improve the latent tuberculosis Cascade of Care in Ghana: a longitudinal impact assessment. BMC Infect Dis. 2020 May 18;20(1):352. eng. Epub 2020/05/20. doi:10.1186/s12879-020-05060-0. Cited in: Pubmed; PMID 32423422.

105. Faust L, Ruhwald M, Schumacher S, Pai M. How are high burden countries implementing policies and tools for latent tuberculosis infection? A survey of current practices and barriers. Health Sci Rep. 2020 Jun;3(2):e158. eng. Epub 2020/05/07. doi:10.1002/hsr2.158. Cited in: Pubmed; PMID 32373716.

106. Hall J, Kabir TM, Shih P, Degeling C. Insights into culturally appropriate latent tuberculosis infection (LTBI) screening in NSW: perspectives of Indian and Pakistani migrants. Aust N Z J Public Health. 2020 Oct;44(5):353-359. eng. Epub 2020/08/11. doi:10.1111/1753-6405.13021. Cited in: Pubmed; PMID 32776658.

107. Salem BE, Klansek E, Morisky DE, Shin SS, Yadav K, Chang AH, Nyamathi AM. Acceptability and Feasibility of a Nurse-Led, Community Health Worker Partnered Latent Tuberculosis Medication Adherence Model for Homeless Adults. Int J Environ Res Public Health. 2020 Nov 11;17(22). eng. Epub 2020/11/15. doi:10.3390/ijerph17228342. Cited in: Pubmed; PMID 33187301.

108. Sharma N, Basu S, Chopra KK, Sharma P. Awareness and perspectives on expansion of latent TB management among public-sector physicians and medical trainees in Delhi, India. Indian J Tuberc. 2020 Apr;67(2):208-212. eng. Epub 2020/06/20. doi:10.1016/j.ijtb.2020.02.003. Cited in: Pubmed; PMID 32553313.

109. Heyd A, Heffernan C, Storey K, Wild TC, Long R. Treating latent tuberculosis infection (LTBI) with isoniazid and rifapentine (3HP) in an inner-city population with psychosocial barriers to treatment adherence: A qualitative descriptive study. PLOS global public health. 2021 2021;1(12):e0000017-e0000017. doi:10.1371/journal.pgph.0000017. Cited in: Pubmed; PMID MEDLINE:36962068.

110. Islam MS, Chughtai AA, Banu S, Seale H. Context matters: Examining the factors impacting the implementation of tuberculosis infection prevention and control guidelines in health settings in seven high tuberculosis burden countries. J Infect Public Health. 2021 May;14(5):588-597. eng. Epub 2021/04/14. doi:10.1016/j.jiph.2021.01.014. Cited in: Pubmed; PMID 33848888.

111. Nathavitharana RR, van der Westhuizen A, van der Westhuizen HM, Mishra H, Sampson A, Meintjes J, Nardell E, McDowell A, Theron G. "If I've got latent TB, I would like to get rid of it": Derivation of the CARD (Constraints, Actions, Risks, and Desires) Framework informed by South African healthcare worker perspectives on latent tuberculosis treatment. PLoS One. 2021;16(8):e0254211. eng. Epub 2021/08/19. doi:10.1371/journal.pone.0254211. Cited in: Pubmed; PMID 34407070.

112. Yanes-Lane M, Trajman A, Bastos ML, Oxlade O, Valiquette C, Rufino N, Fregonese F, Menzies D. Effects of programmatic interventions to improve the management of latent tuberculosis: a follow up study up to five months after implementation. BMC Public Health. 2021 Jan 21;21(1):177. eng. Epub 2021/01/23. doi:10.1186/s12889-021-10195-z. Cited in: Pubmed; PMID 33478452.

113. Gray AT, Surey J, Esmail H, Story A, Harris M. "It's too hard" - the management of latent TB in under-served populations in the UK: a qualitative study. BMC Health Serv Res. 2022 Dec 1;22(1):1464. Epub 2022/12/03. doi:10.1186/s12913-022-08855-w. Cited in: Pubmed; PMID 36457026.

114. Gustavson G, Narita M, Gardner Toren K. Reporting of Latent TB Infection Among Non-US-Born Persons Adjusting Their Immigration Status to Permanent Residents: An Opportunity to Enhance TB Prevention. J Public Health Manag Pract. 2022 Mar-Apr 01;28(2):184-187. eng. Epub 2021/08/05. doi:10.1097/phh.0000000000001405. Cited in: Pubmed; PMID 34347651.

115. Szkwarko D, Kim S, Carter EJ, Goldman RE. Primary care providers' and nurses' knowledge, attitudes, and skills regarding latent TB infection testing and treatment: A qualitative study from Rhode Island. PLoS One. 2022;17(4):e0267029. Epub 2022/04/16. doi:10.1371/journal.pone.0267029. Cited in: Pubmed; PMID 35427377.

116. An Y, Teo AKJ, Huot CY, Tieng S, Khun KE, Pheng SH, Leng C, Deng S, Song N, Nonaka D, Yi S. They do not have symptoms - why do they need to take medicines? Challenges in tuberculosis preventive treatment among children in Cambodia: a qualitative study. BMC Pulm Med. 2023 Mar 10;23(1):83. eng. Epub 2023/03/11. doi:10.1186/s12890-023-02379-7. Cited in: Pubmed; PMID 36899328.

117. Carter J, Knights F, Deal A, Crawshaw AF, Hayward SE, Hall R, Matthews P, Seedat F, Ciftci Y, Zenner D, Wurie F, Campos-Matos I, Majeed A, Requena-Mendez A, Hargreaves S. Multi-infection screening for migrant patients in UK primary care: Challenges and opportunities. J Migr Health. 2024;9:100203. eng. Epub 2023/12/07. doi:10.1016/j.jmh.2023.100203. Cited in: Pubmed; PMID 38059072.

118. H SNF, Manoharan A, Koh WM, K M, Khoo EM. Facilitators and barriers to latent tuberculosis infection treatment among primary healthcare workers in Malaysia: a qualitative study. BMC Health Serv Res. 2023 Aug 29;23(1):914. Epub 2023/08/30. doi:10.1186/s12913-023-09937-z. Cited in: Pubmed; PMID 37644513.

119. Manoharan A, Siti Nur Farhana H, Manimaran K, Khoo EM, Koh WM. Facilitators and barriers for tuberculosis preventive treatment among patients with latent tuberculosis infection: a qualitative study. BMC Infect Dis. 2023 Sep 22;23(1):624. Epub 2023/09/23. doi:10.1186/s12879-023-08612-2. Cited in: Pubmed; PMID 37740196.

120. Salles I, Travassos P, Spener-Gomes R, Loch AP, Saraceni V, Lauria L, Cavalcante S, Garcia de Oliveira J, Brito de Souza A, Guimaraes Costa A, Sakabe S, Schiavon Nogueira R, Chaisson LH, Cohn S, Jamal LF, Valdez Ramalho Madruga J, Cordeiro-Santos M, Castro B, Portella Ferreira D, Hoffmann CJ, Golub JE, Durovni B, Kerrigan D. Contextualizing and optimizing novel strategies to improve the latent TB continuum of care: Insights from people living with HIV and health care providers in Brazil. PLOS global public health. 2023 2023;3(1):e0001251-e0001251. doi:10.1371/journal.pgph.0001251. Cited in: Pubmed; PMID MEDLINE:36962892.

121. Shamputa IC, Law MA, Kelly C, Nguyen DTK, Burdo T, Umar J, Barker K, Webster D. Tuberculosis related barriers and facilitators among immigrants in Atlantic Canada: A qualitative study. PLOS Glob Public Health. 2023;3(6):e0001997. eng. Epub 2023/06/05. doi:10.1371/journal.pgph.0001997. Cited in: Pubmed; PMID 37276222.

122. Spence BC, Bruxvoort K, Munoz-Plaza C, Shaw S, Navarro M, Chen H, Skarbinski J, Tartof S. Patient-Reported Barriers to Treatment Initiation and Completion for Latent Tuberculosis Infection Among Patients Within a Large Integrated Health Care System in Southern California. J Public Health Manag Pract. 2023 May-Jun 01;29(3):345-352. Epub 2023/03/04. doi:10.1097/PHH.0000000000001711. Cited in: Pubmed; PMID 36867508.

123. Giridharan P, Suseela RP, Zangpo T, Joshi RB, Cader M, Isbaniah F, Velayudham B, Rafeeg FN, da Cruz Santos A, Shah NP, Mathew M, George LS, Gupta N, Padmapriyadarsini C. Tuberculosis preventive treatment in eight SEAR countries - Current practices, implementation challenges and operations research priorities. Public Health Pract (Oxf). 2024 Dec;8:100518. eng. Epub 2024/07/24. doi:10.1016/j.puhip.2024.100518. Cited in: Pubmed; PMID 39045000.

124. Atchison C, Zenner D, Barnett L, Pareek M. Treating latent TB in primary care: a survey of enablers and barriers among UK General Practitioners. BMC Infect Dis. 2015 Aug 13;15:331. eng. Epub 2015/08/14. doi:10.1186/s12879-015-1091-9. Cited in: Pubmed; PMID 26268227.

125. Walker CL, Duffield K, Kaur H, Dedicoat M, Gajraj R. Acceptability of latent tuberculosis testing of migrants in a college environment in England [Article]. Public Health. 2018;158:55-60. English. doi:10.1016/j.puhe.2018.02.004.

126. Nwana N, Marks SM, Lan E, Chang AH, Holcombe M, Morris SB. Treatment of latent Mycobacterium tuberculosis infection with 12 once weekly directly-observed doses of isoniazid and rifapentine among persons experiencing homelessness. PLoS One. 2019;14(3):e0213524. eng. Epub 2019/03/14. doi:10.1371/journal.pone.0213524. Cited in: Pubmed; PMID 30865724.

127. Spruijt I, Erkens C, Suurmond J, Huisman E, Koenders M, Kouw P, Toumanian S, Cobelens F, van den Hof S. Implementation of latent tuberculosis infection screening and treatment among newly arriving immigrants in the Netherlands: A mixed methods pilot evaluation. PLoS One. 2019;14(7):e0219252. eng. Epub 2019/07/02. doi:10.1371/journal.pone.0219252. Cited in: Pubmed; PMID 31260502.

128. Spruijt I, Tesfay Haile D, Suurmond J, van den Hof S, Koenders M, Kouw P, van Noort N, Toumanian S, Cobelens F, Goosen S, Erkens C. Latent tuberculosis screening and treatment among asylum seekers: a mixed-methods study. Eur Respir J. 2019 Nov;54(5). eng. Epub 2019/09/21. doi:10.1183/13993003.00861-2019. Cited in: Pubmed; PMID 31537698.

129. Ngugi SK, Muiruri P, Odero T, Gachuno O. Factors affecting uptake and completion of isoniazid preventive therapy among HIV-infected children at a national referral hospital, Kenya: a mixed quantitative and qualitative study. BMC Infect Dis. 2020 Apr 21;20(1):294. eng. Epub 2020/07/16. doi:10.1186/s12879-020-05011-9. Cited in: Pubmed; PMID 32664847.

130. Spruijt I, Haile DT, Erkens C, van den Hof S, Goosen S, Ten Kate A, Teshome H, Karels M, Koenders M, Suurmond J. Strategies to reach and motivate migrant communities at high risk for TB to participate in a latent tuberculosis infection screening program: a community-engaged, mixed methods study among Eritreans. BMC Public Health. 2020 Mar 12;20(1):315. eng. Epub 2020/03/14. doi:10.1186/s12889-020-8390-9. Cited in: Pubmed; PMID 32164637.

131. Kunin M, Timlin M, Lemoh C, Sheffield DA, Russo A, Hazara S, McBride J. Improving screening and management of latent tuberculosis infection: development and evaluation of latent tuberculosis infection primary care model. BMC Infect Dis. 2022 Jan 12;22(1):49. Epub 2022/01/14. doi:10.1186/s12879-021-06925-8. Cited in: Pubmed; PMID 35022023.

132. Chevrier C, Diaz MH, Rueda ZV, Balakumar S, Haworth-Brockman M, Marin DM, Oliver A, Plourde P, Keynan Y. Introduction of short course treatment for latent tuberculosis infection at a primary care facility for refugees in Winnipeg, Canada: A mixed methods evaluation. Front Public Health. 2022;10:1064136. eng. Epub 2023/02/03. doi:10.3389/fpubh.2022.1064136. Cited in: Pubmed; PMID 36726628.

133. Alvi Y, Philip S, Anand T, Chinnakali P, Islam F, Singla N, Thekkur P, Khanna A, Vashishat BK. Situation Analysis of Early Implementation of Programmatic Management of Tuberculosis Preventive Treatment among Household Contacts of Pulmonary TB Patients in Delhi, India. Trop Med Infect Dis. 2024 Jan 17;9(1). eng. Epub 2024/01/22. doi:10.3390/tropicalmed9010024. Cited in: Pubmed; PMID 38251221.

134. Barroso E, Mark T, Acevedo R, Rao S, Jordan HT, Burzynski J, Remegio W, Ea E, Compas L. Patient navigator's role in latent tuberculosis infection at a New York City Health Department Chest Clinic. J Clin Tuberc Other Mycobact Dis. 2024 Aug;36:100446. eng. Epub 2024/05/06. doi:10.1016/j.jctube.2024.100446. Cited in: Pubmed; PMID 38708035.
